# Trellis for efficient data and task management in the VA Million Veteran Program

**DOI:** 10.1038/s41598-021-02569-5

**Published:** 2021-12-01

**Authors:** Paul Billing Ross, Jina Song, Philip S. Tsao, Cuiping Pan

**Affiliations:** 1 Stanford Center for Genomics and Personalized Medicine, Stanford University, Stanford, CA USA; 2 Department of Medicine, Stanford University, Stanford, CA USA; 3 Palo Alto Epidemiology Research and Information Center for Genomics, VA Palo Alto, CA USA

**Keywords:** Computational biology and bioinformatics, Data processing, Databases, Genome informatics, Software

## Abstract

Biomedical studies have become larger in size and yielded large quantities of data, yet efficient data processing remains a challenge. Here we present Trellis, a cloud-based data and task management framework that completely automates the process from data ingestion to result presentation, while tracking data lineage, facilitating information query, and supporting fault-tolerance and scalability. Using a graph database to coordinate the state of the data processing workflows and a scalable microservice architecture to perform bioinformatics tasks, Trellis has enabled efficient variant calling on 100,000 human genomes collected in the VA Million Veteran Program.

## Introduction

The surge of biomedical data in the last decade has propelled a deeper understanding of how genetic variants contribute to healthy and diseased states in humans. Large studies involving 1000 s to 100,000 s of study participants have been essential to understanding these mechanisms^1–5^. The volumes of data generated and the subsequent computation are massive. For example, the GATK pipeline for processing whole genome sequencing data contains 16 steps, with some steps sharding data into 50 partitions for parallel computing, resulting in ~ 1000 data objects and 150 jobs for a single genome^6^. When this scales out to thousands of genomes, millions of data objects and jobs will be generated. Effectively managing such large-scale data is essential to knowledge discovery and innovation.

The FAIR (Findable, Accessible, Interoperable, Reproducible) principles for scientific data management and stewardship provide a useful set of guidelines for managing such data, but implementing them remains a significant challenge^7^. In addition, these challenges are compounded for long-term research programs that increase in complexity over time as they evolve to incorporate new data and methods. To overcome these challenges, we have developed a cloud-based data management framework, Trellis, that uses a graph database to automatically track data and coordinate jobs, in accordance with FAIR data principles. We applied it to call variants on 100,000 human genomes in the VA Million Veteran Program (MVP)^8^ and found that it streamlined data processing, reduced operational overhead, and achieved robust performance over the course of several months of continuous processing. This work demonstrates that Trellis can improve the pace and quality of scientific research in large biomedical studies.

## Results

### The design of Trellis

**Figure 1:**
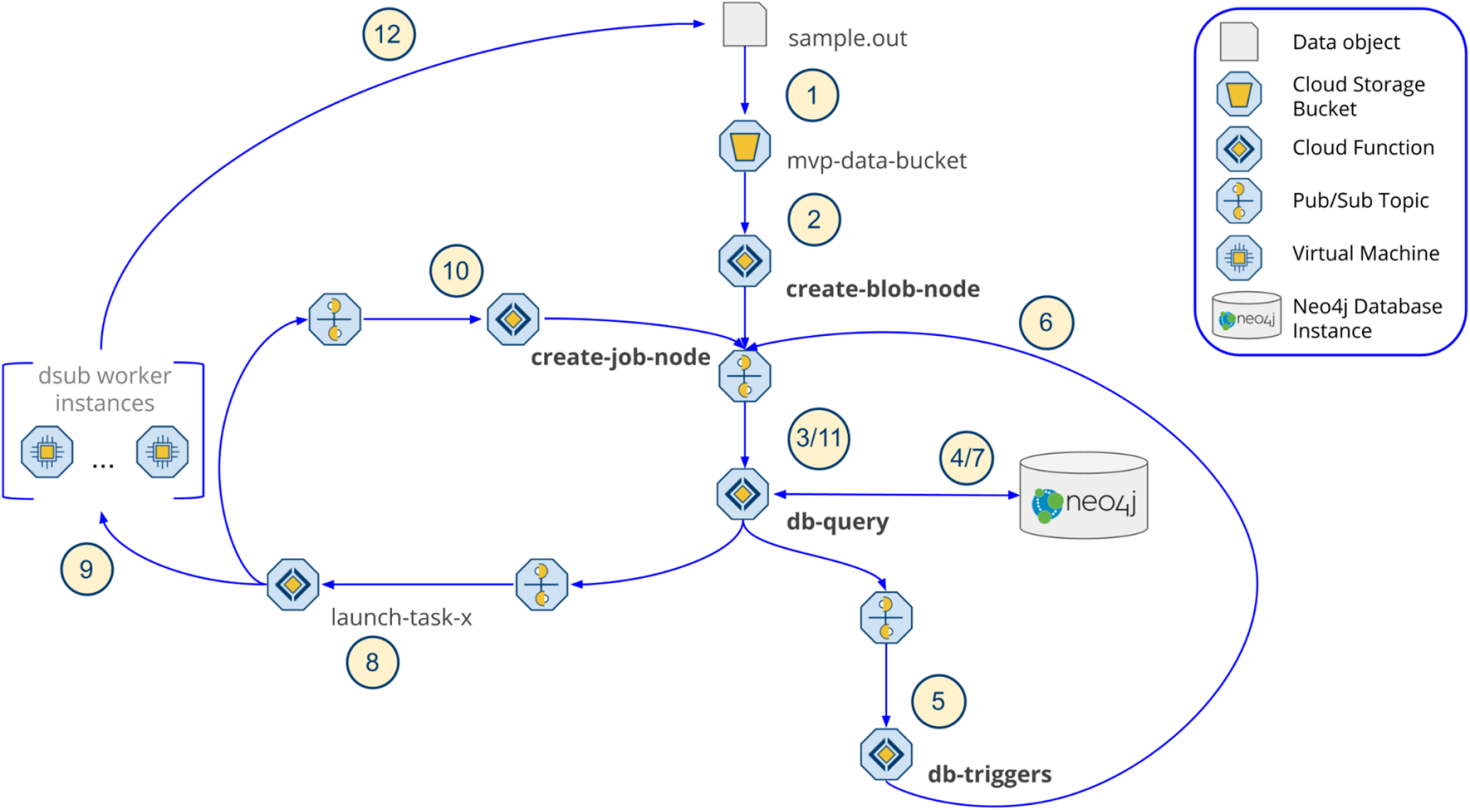
In Trellis, services are implemented as serverless functions that communicate with each other via message brokers. The state of the system is tracked using metadata stored in a graph database. This architecture is responsible for representing data objects as nodes in the database, checking their metadata against database triggers, launching jobs, and representing the jobs as nodes in the database. Users interact with Trellis by submitting queries to a GraphQL API. The standard operating cycle for tracking data and launching jobs starts when a data object is transferred into a storage bucket tracked by Trellis. The metadata for the object is used to create a reference to the object as a node in the database. The properties of that node are compared against all of the database triggers and launch operations appropriate for processing the data object. If the operation is a bioinformatics task, it will be launched by a function specific to that task. The resulting outputs of the job(s) will be added to a bucket and the cycle continues.

Trellis uses cloud-native technologies optimized for massively parallel operations. The application follows a microservice architecture where each service is implemented as a serverless function and the state of the system is tracked using metadata stored in a Neo4j property graph database (Fig. 1). The value of using a graph database is that metadata can be natively modelled according to the structure of a bioinformatics workflow, while the microservice architecture makes it easy to add or remove services for different needs.

The Trellis application logic is implemented in serverless functions so that it can automatically scale to meet the demands of large-scale analyses. Serverless functions are short pieces of code that define the logic to perform a single operation. The execution of serverless functions is handled by the cloud provider, so there is no need to provision servers or configure auto-scaling policies. They are useful for parallelizing small operations across distributed computing systems because each function runs independently, without relying on stateful information from other parts of the system^9^. This allows Trellis services to rapidly scale up or down, across multiple machines, to meet the demands of thousands of jobs running simultaneously. Because they are stateless, the services use a message broker to communicate with each other and register all system updates in the graph database. The implementation of Trellis relies on cloud technologies, which can be either open-source or serviced. A few example products for each module are listed in Table 1.

**Table 1:** Examples of open-sourced or managed services to implement the primary components of the Trellis model on both public and private cloud platforms.

Component	Open-source product	Serviced product
Database	Community edition of Neo4j	Enterprise edition of Neo4j
Function as a service (serverless functions)	Kubernetes OpenFaaS	Cloud functions (GCP) Lambda (AWS)
Container as a service	Knative	Cloud run (GCP) Fargate (AWS)
Message broker	Apache Kafka	Pub/sub topics (GCP)
Job monitoring	Prometheus	StackDriver (GCP)
Summary statistics representation	JupyterLab	Data studio (GCP)

Trellis relies on four core functionalities that work in tandem to manage data and jobs (Supplementary Fig. 1). The first is to represent data objects as nodes in the graph database. This occurs when data objects are added to or updated in a cloud storage bucket. The next step determines whether any processing needs to be performed on the object. This is done by comparing the metadata of the object against the conditions for triggering processing tasks. If a task is triggered, the third step is to query the database for the job inputs and send them to a function to launch it. Finally, once the job has been launched, it is recorded as a node in the database. These four functionalities represent the core loop used by Trellis to manage and track all data objects and computing tasks in a graph database, which can be queried for the purposes of finding relevant data or process optimization. In the following sections, we expound upon these key functionalities that can be expanded to support data management for a broad range of data processing workflows.

#### Creating nodes for data objects

Trellis is an event-driven system where the primary events are new data objects being added to a storage bucket that is monitored by Trellis (Fig. 1, step 1). Creation of a data object activates a cloud storage trigger which sends the metadata of the object to the create-blob-node function (Fig. 1, step 2). The function parses the object metadata from its cloud storage path, or an associated JSON object, and creates a database query that will add a reference to the object as a node in the database. It sends the query via a message broker, such as Cloud Pub/Sub, to the db-query function which will communicate it to the Neo4j graph database (Fig. 1, step 3).

Neo4j is a labelled property graph, where nodes and relationships can be labelled with strings and populated with key:value properties. We treated labels as categories of entities where each label corresponds to a particular set of properties. In cases where an object could fit multiple categories, we assigned multiple labels to that node. For instance, nodes representing Fastq objects were assigned the labels “Blob” and “Fastq”. The “Blob” label corresponds to properties of blobs/objects stored in cloud storage such as object path, storage bucket, size, and crc32c hash value. The size value can be used to predict computational requirements of downstream processing steps while the hash value can establish data fidelity. The “Fastq” label is associated with experimental properties such as the sequencing plate identifier, sequencing read group, and the mate pair index in the case of paired-end sequencing. These values can help identify and correct for batch effects from experimental artifacts. We used the Bento Graph Model Description Framework^10^, developed at the National Cancer Institute Center for Biomedical Informatics & Information Technology, to define our data model (Supplementary List 1, Supplementary Fig. 2). In doing so, we aimed to provide a clearly structured and user-friendly description of the data relationships that will facilitate future integration with other large biomedical databases.

#### Activating database triggers

Trellis automatically launches jobs based on metadata. When a node is added or updated in the database, the database query returns the properties of the node to the db-query function (Fig. 1, step 4). These metadata properties are sent to the db-triggers function to determine whether any subsequent operations should be initiated (Fig. 1, step 5). The operations of Trellis are controlled by database triggers which are managed by db-triggers. These operations consist primarily of two steps, adding relationships between nodes in the database and triggering bioinformatics tasks. Each trigger has two functional components, that is, a set of metadata conditions that must be met to activate the trigger, and a database query that will run on activation to get the inputs for the triggered operation. When node properties are received, they are checked against the conditions for activating each trigger. If the conditions are met, the corresponding database query is sent to db-query to be processed by the database (Fig. 1, step 6).

#### Running database trigger queries

**Figure 2:**
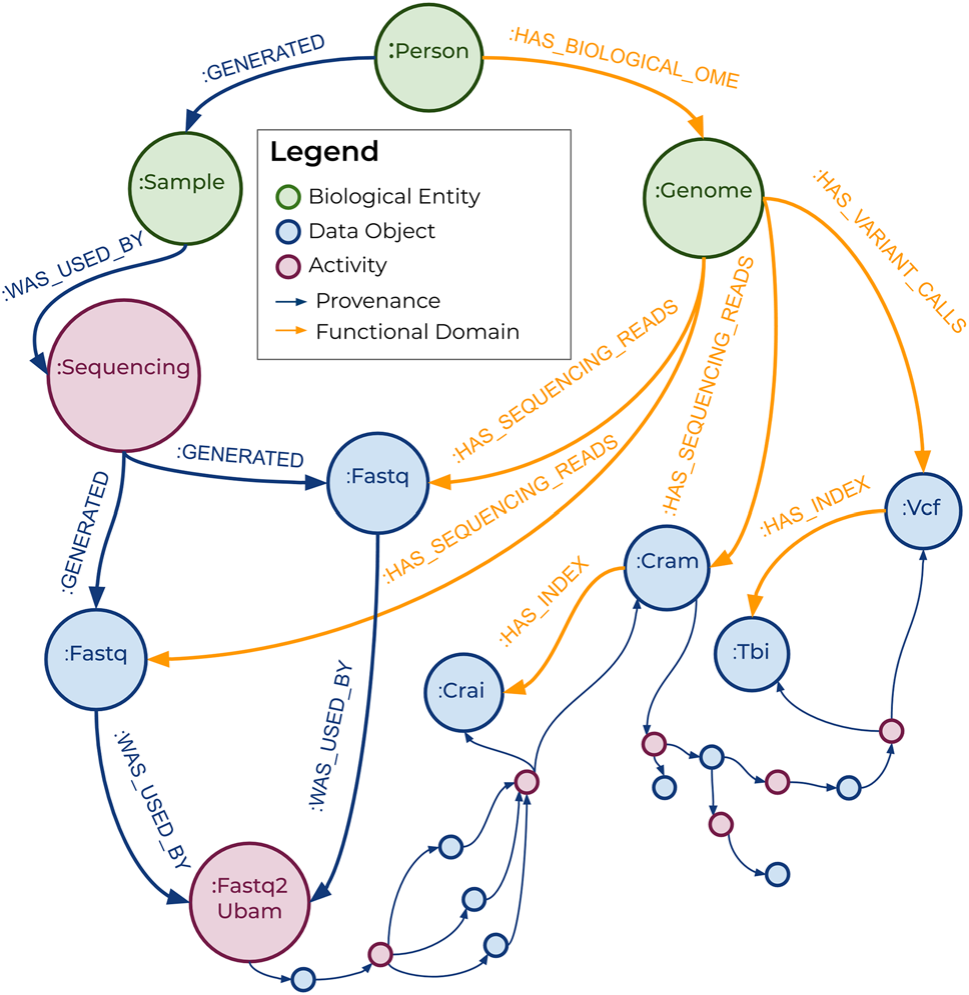
The graph database model includes different types of nodes that are connected by two categories of relationships: provenance domain and functional domain. These relationships allow for querying of data that is related via lineage or functional relevance.

The graph database acts as the application hub, tracking metadata and controlling Trellis operations. Whereas the conditions for activating a database trigger determine whether the content of a node is right for launching an operation, the trigger query determines whether the context of the node is appropriate (“Methods”). The database includes nodes representing data objects and jobs as well as biological entities such as people, molecular samples, and the genome. Nodes are connected using two relationship patterns, one that describes data provenance (blue relationships in Fig. 2), and one that relates data to functional research domains (orange relationships in Fig. 2). Provenance relationships are designed to improve reusability of data by clearly tracing the lineage of every data object to a biological sample of origin. Functional domain relationships are designed to simplify the process of querying data most relevant to a particular research domain; in this case, the human genome. Trigger queries traverse these relationships to determine whether the graph state meets the conditions for launching an operation (Fig. 1, step 7). If the conditions are met, the query returns the inputs necessary to launch the job and the db-query function sends them to the appropriate launcher function (Fig. 1, step 8).

**Figure 3:**
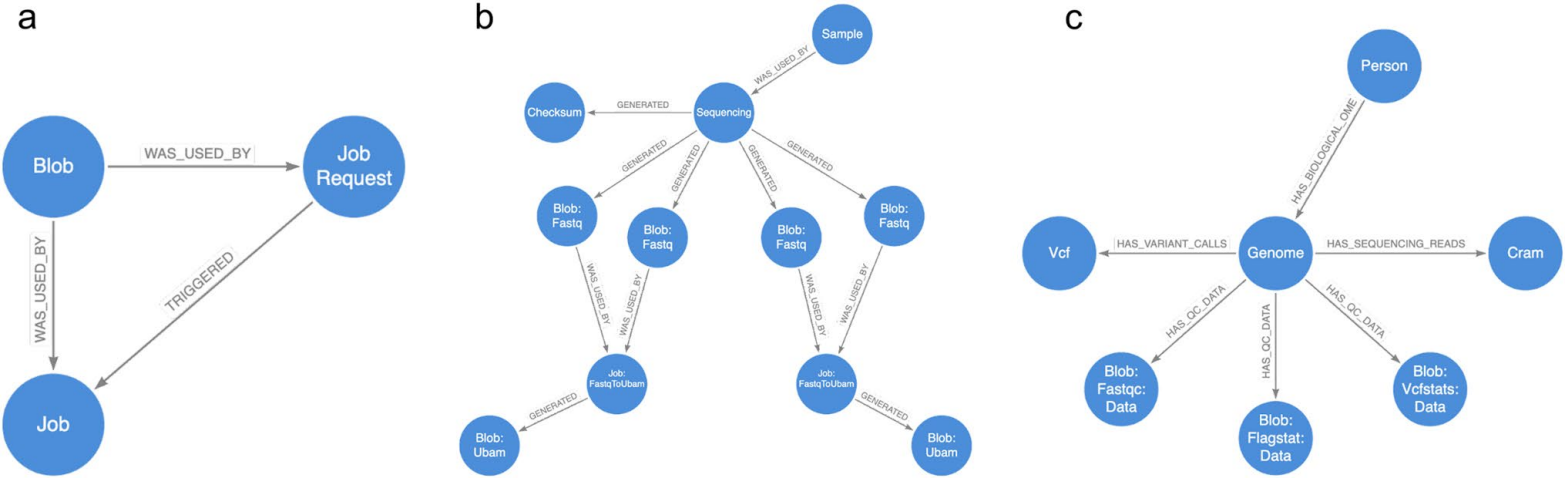
Use case of the graph data model in Trellis. (**a**) Job request nodes are used to prevent jobs being launched in duplicate. (**b**) The provenance relationships can be used to query all the unaligned bams generated for a sample, and return them as inputs for variant calling. (**c**) Relationships used to describe the functional relationships of data to the “genome” domain, are used to query that all essential data types have been generated for a sample, before deleting intermediate objects.

Triggers use relationships to serve a variety of purposes. To avoid launching duplicate jobs, queries for launching bioinformatics tasks check for existing job request nodes before requesting a job (Fig. 3a). Provenance relationships are used to query that an unaligned BAM object has been generated for each sequencing group, before launching GATK variant calling. The query finds all the unaligned BAMs belonging to unique read groups and compares that number to a property stored in the delivery manifest/checksum node, to ensure that all groups are accounted for (Fig. 3b).

Because we store data indefinitely on the cloud, storage represents our biggest cost. And as the intermediate objects generated during variant calling often exceed 300 GB in size, we implemented a function to delete these objects after variant calling. Before initiating this process, the trigger query starts from the genome node and traverses functional domain relationships to determine that all essential genomic data types (Cram, gVCF, FastQC, Flagstat, Vcfstats) have been generated (Fig. 3c). In this way, different relationship patterns increased the connectedness of our data and improved our ability to automatically make functional decisions about how to process them.

#### Creating job nodes

Each bioinformatics task is initiated by a different function, whose single purpose is to launch that task. Trellis configures the jobs and relies on separate task/workflow managers to handle running them (i.e. provisioning virtual machines, running the bioinformatics application(s), transferring input/output objects). The function takes the inputs specified from the database query results, transforms them into a job configuration, and submits them to the task manager (Fig. 1, step 9). We used the Docker-based Dsub task manager to run QC jobs and Cromwell to run the germline variant-calling pipeline. We chose Cromwell for the latter, because the $5 GATK pipeline that we used for variant calling was already defined in Cromwell’s native Workflow Definition Language (WDL) and the pipeline had already been optimized to run on Google Cloud using Cromwell. Using Dsub and Cromwell, we achieved a 99.9% success rate for Fastq-to-ubam jobs and > 98% success rate for GATK pipelines jobs (Supplementary Tables 6, 7).

Once a job has been submitted, the launcher sends the configuration data to the create-job-node function so that it can be added as a node in the database database (Fig. 1, step 10). Job nodes include all the information necessary to replicate them, such as the Docker image^11^, computing environment configuration, input path(s), and command used to run the job. These metadata are translated into node properties and incorporated into a node creation query that is sent to the db-query function to be processed (Fig. 1, step 11). Once the job node has been added to the database, it will activate triggers to relate the node to the object nodes that were used as inputs. Similarly, as outputs are generated from the job, they will be linked to the job node via provenance relationships.

#### Interacting with the graph database

Interaction with the database is mediated via a GraphQL application programming interface (API) that, like the Neo4j database, models data graphically^12^. GraphQL is part of the GRANDstack^13^ application framework that is designed for building graph applications, and as such, we found a lot of developer resources for integrating it with Neo4j. The API serves as a contract between the user and the database, defining what types of data are queryable and how those requests will be resolved by the database. This layer of control protects data integrity and ensures that only approved users have access to sensitive data. Because the contract is defined using a schema that decouples data types from resolvers, the same API requests can query databases that use different models or technologies. In this way, the API provides a consistent mechanism for querying data and an interoperable template for implementing the Trellis data model in other database environments.

#### Security

Maintaining data security is always important when handling genomic data which has the potential to identify people and their phenotypes. In building Trellis, we followed the security guideline for computing genomics data on the cloud^14^. In addition, we have tried to minimize security risk by running Trellis on a virtual private cloud (VPC) network (Supplementary Fig. 3). Virtual machines operate with only internal IP addresses and serverless functions communicate using an internal message broker to avoid exposing HTTP endpoints. The Neo4j database has an external IP address to allow developer access, but this access is tightly controlled through IP-specific firewall rules. With respect to the overall computing environment, Google Cloud, where Trellis is currently implemented, is compliant with HIPAA, FEDRAMP, and FISMA-moderate Authorization, and data on Google Cloud is encrypted at all times.

### Trellis for WGS processing in MVP

We deployed Trellis to Google Cloud Platform (GCP)^14^ for performing germline variant calling and quality assessment on whole-genome sequencing data from MVP (Supplementary Table 1)^15–18^. Trellis configures jobs based on database metadata and sends the configuration to a separate job or workflow manager. In this case, we used Cromwell^19^ to run the GATK pipeline to call single nucleotide variants and insertion-deletion variants, and used Dsub^20^ to run pre-processing and quality assessment. All jobs were run on virtual machines in an isolated virtual private cloud environment to ensure secure handling of genomes (“Methods”) (Supplementary Fig. 3). We leveraged cloud logging service StackDriver to monitor jobs and the visualization dashboard Data Studio to present summary statistics of the workflows and results (“Methods”) (Supplementary Fig. 4).

**Figure 4:**
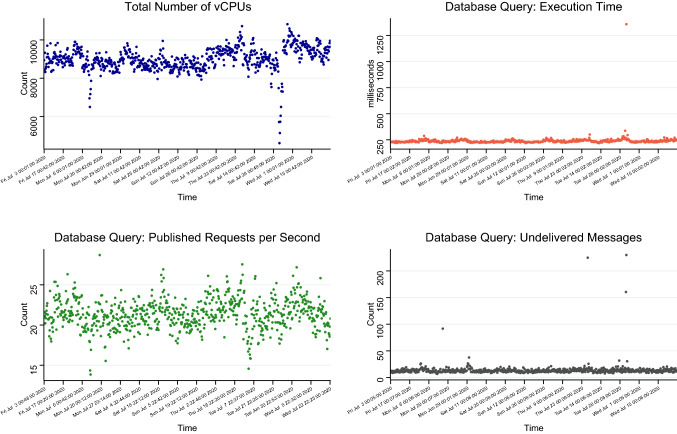
Performance of the Trellis model of processing human genomes, including GATK variant calling and quality assessment, at the speed of 600 genomes a day. The data presented came from processing 100,000 genomes in the Million Veteran Program. (**Top left**) Total number of vCPUs used. These included all vCPUs used by Trellis, including for variant calling and QC, as well as for running the database and Jupyter analysis notebook. (**Top right**) Execution times for database query functions. They remained constant at about 200 ms/query . This demonstrated that queries were well optimized and the database remained performant as load fluctuated. (**Bottom left**) Number of messages published to the database query function per second. At the peak times, about 20 database queries messages were sent out per second, reading the maximum capacity that the current architecture of Trellis was able to handle. (**Bottom right**). Number of undelivered messages. Rarely were more than 20 messages undelivered.

As mentioned previously, the functional equivalence GATK pipeline generated around ~ 1,000 data objects and 150 computational tasks for a single 30 × coverage genome, and this amounted to about 100 million data objects and 15 million tasks from processing the 100,000 whole genomes in MVP (Supplementary Fig. 5). Running an end-to-end variant calling and quality control workflow for a single sample took an average of 802 CPU hours (Supplementary Fig. 6, Supplementary List 2). With Trellis, we routinely processed 600 genomes a day, using about 6,000 preemptible vCPUs for the GATK tasks, and an additional 1,500 standard vCPUs to host Cromwell controllers, preprocess data (i.e. Fastq-to-ubam) and assess data quality (Fig. 4). These operations were robust enough to achieve a 99.9% and 98.4% average daily success rates for the Fastq-to-ubam presprocessing and the GATK workflow, respectively (Supplementary Figs. 7, 8). Furthermore, our system reduced duplicate jobs to the minimal level (Supplementary Figs. 9–12).

Detailed cost analysis revealed that 96.3% of the compute cost was spent on bioinformatics tasks (i.e. virtual machines), while 2% went to job monitoring and only 1% was spent on operating Trellis (Supplementary Fig. 13) (Supplementary Table 2). Although we provided genomic analysis as an example application, the Trellis framework can be expanded to accommodate various workflows by following the design principles described here.

After processing, data is stored indefinitely in cloud storage. To reduce store costs, we initiate data purging and archival processing as soon as all essential data types have been generated. This involves (1) moving Fastq objects from standard storage to cold line storage and (2) deleting all the intermediate objects generated during variant calling. After this, the total storage footprint is about 65 GB per sample and costs roughly $0.5/sample/month to keep in Google Cloud Storage (Supplementary Table 3).

## Discussion

Large consortia studies, such as VA MVP^8^, gnomAD^1^, and TOPMed^2^, combine resources of different organizations for consolidating large and high quality datasets and can run for years, releasing multiple versions of data generated from varied experimental conditions, softwares, and reference materials. To organize and manage these biomedical data in accordance with the FAIR principles is essential for efficient data analysis, result validation and enabling a broad spectrum of downstream research. Our data and task management system, Trellis, provides a scalable solution to automatically track data and coordinate workflows in large biomedical studies. Our application of Trellis to calling 100,000 whole genomes in the VA Million Veteran Program has observed robust and consistent high performance throughout the process. Using an event-driven model, it was able to operate automatically, with minimal human oversight. In addition, it added only a marginal cost to the analysis pipeline for job monitoring (2%) and even smaller (1%) to operating Trellis itself.

Trellis achieved this performance by leveraging managed services widely available on cloud computing platforms. The program logic was implemented via serverless functions to fit a microservice architecture that makes it easy to add additional application services when the scope of the data types and analyses change over the course of a project lifetime. Trellis leveraged database triggers to automatically launch jobs when appropriate start conditions were met and was also able to support fault tolerance by including triggers to automatically respond to failure cases. Furthermore, by using preemptible VMs we were able to further reduce our cloud computing costs.

In the Trellis database, the connections between data, jobs, and biological concepts are modelled as graphs. Just as all the systems in a living body are connected, so are the data collected from those bodies. Increasing the connectivity between those data makes it easier to reproduce results as well as find information relevant to particular research questions. We mapped these connections in two ways. For one, we connected data objects and jobs sequentially, in the order they were generated by bioinformatics workflows. This provides a mechanism for retrieving the data lineage of each object and the metadata required to reproduce the workflow used to generate it. The other method connects objects to concepts in a biological domain, such as the genome. These relationships establish the functional relevance of the object and enable querying subsets of data relevant to a particular research question. This functionality is suited to big data, where analyzing an entire dataset may not be feasible, as well as to sharing sensitive biomedical data in a manner that is responsible and useful. For integrative research that span multiple biological domains or organisms, the Trellis database model can be expanded to accommodate various data types and their metadata.

The framework provided by Trellis can support various bioinformatics workflows. We applied Trellis to manage the variant calling and quality-control of DNA sequencing reads. Using the same design concepts, we are now expanding Trellis to support more genomics workflows, including comprehensive structural variant calling, somatic variant calling, human leukocyte antigen typing, etc.

We also note that the rich metadata stored in the Trellis database provides a central hub for cohort building. Because the database does not enforce schema constraints, it supports disparate entity types, making it possible to include biological concepts such as genes and chromosomes, clinical or phenotypic conditions, as well as journal publications, all in the same database. This is particularly valuable for biobank designs where different study cohorts are formed by permutating samples based on their phenotypes or experimental conditions. A dynamic and interactive database, with rich metadata populated from across research domains, will no doubt facilitate cohort building, and expand the Trellis applications for large biomedical studies.

## Methods

### Overview of Trellis implementation

Trellis leverages a graph database and microservices to manage data objects and computational tasks. We implemented it on GCP chiefly using a virtual machine instance for the Neo4j graph database and three managed services: Cloud Functions, Cloud Pub/Sub, and Cloud Run. The application logic was encoded in serverless functions that run in the Cloud Functions or Cloud Run execution environments. Functions communicate with each other, and the database, using messages transmitted by the Cloud Pub/Sub message broker. Most functions, which only contained Python code in our implementation, were run using Cloud Functions. Ones that required additional resources, such as compiled programs, were run using Cloud Run. In the following, we focused on our Trellis implementation on GCP for running genomics workflows for the MVP. However, it is worth pointing out that both open-sourced and paid services are available for each category of technology we used, therefore Trellis can be implemented in various computing systems (Table 1).

In the following, we describe the Trellis methods applied to the WGS data processing in MVP. Recruitment and sample collection for the MVP received ethical and study protocol approval by the Veterans Affairs Central Institutional Review Board in accordance to the principles outlined in the Declaration of Helsinki and approved by the R&D Committee of the VA Palo Alto Health Care System. All methods were carried out in accordance with the VA and Stanford IT guidelines and regulations.

### Genomics workflows

For detecting DNA variants in the human genomes sequenced in MVP, we adopted the $5 GATK pipeline from the Broad Institute^6^, which complied with the functional equivalence advocated by other large organizations^15^. Briefly, the human reference genome GRCh38 was used for mapping and variant calling. Alignment was performed by BWA-MEM (v0.7.15) with the command line: bwa mem -K 100,000,000 -p -v 3 -t 16 -Y $bash_ref_fasta. PCR duplicates were marked by Picard 2.15.0 and BQSR were performed in GATK 4.1.0.0, resulting in four-bin quality score compression. The alignment results in BAM were compressed to CRAM in a lossless manner. Variant calling was performed in GATK 4.1.0.0 using the haplotypeCaller function, resulting in gVCF files which contained reference call blocks, single nucleotide variants (SNVs), and short insertion and deletions (INDELs). We assessed data quality at multiple levels. On sequencing, we used FastQC (v0.11.4)^16^ to compute base sequence quality, read quality, and GC contents; on read alignments, we used SAMTools flagstat (v0.1.19)^17^ to calculate the number and percentage of properly mapped reads and properly paired reads; on variant calling results, we used RTG Tools vcfstats (v3.7.1)^18^ to count the number of SNVs and Indels per genome, ratio of SNV transition to transversion, and ratio of SNV heterozygous genotypes to homozygous genotypes. In addition, we used verifybamID in GATK 4.1.0.0 to estimate DNA contamination rates for individual genomes. We used Cromwell to manage the GATK workflows, and Dsub to manage the data quality assessment tools. Docker images for these tools are available in Dockerhub (Supplementary Table 4).

### Categories of serverless functions

We designed six categories of functions in Trellis to manage workflows: core functions, job launchers, job monitoring, object management, data import, and extract-transform-load (ETL) (Supplemental Table 5). Core functions were responsible for translating new data objects and jobs into graph nodes, updating the database, and checking database triggers. Job launchers were wrapper functions that took metadata from the database, translated them into a Dsub^20^ command, and ran the job scheduling command. They then sent the metadata to a core function to add the job metadata into the database. Job monitoring functions retrieved data from the GCP Stackdriver logging system to keep track of runtime information for the virtual machines that ran the jobs. The biggest cost sink on the cloud was storage, and the germline variant calling workflow generated 100 s of gigabytes of intermediate data objects in the process of outputting a final VCF. Object management functions were responsible for reducing storage costs after variant calling by deleting intermediate data and moving other objects to more cost-effective storage classes. In the cases where static data already existed in cloud storage, we used the data import functions to add those objects to the database. And extract-transform-load functions were used to transform data from cloud storage objects into other formats. Right now we only used one ETL function for importing quality-control data from individual samples into a PostgreSQL database for calculating population-level quality-control metrics.

### Database configuration

We provisioned a 4-vCPU 140 GB virtual machine running on Intel Broadwell CPUs to run the open-sourced Neo4j database. Due to the limitation of the community version of Neo4j, our database used only 4 vCPUs, but we leveraged a generous memory pool to accommodate database indices and avoid performance bottlenecks. The database runs Neo4j v3.5.14 using the community docker image, retrieved from the official Docker Hub repository (https://hub.docker.com/_/neo4j). When launching the container, the value of the following environment variables were set to “32 G”: NEO4J_dbms_memory_pagecache_size, NEO4J_dbms_memory_heap_initial__size, and NEO4J_dbms_memory_heap_max__size. Resources for the managed services were automatically provisioned by the cloud platform. For most functions, it was not necessary to do much configuration finetuning. However, because the CPU cores limited the number of database queries that could be run in parallel without degrading query performance, the maximum number of db-query instances running at one time was limited to 20.

All of the database information was stored on a disk that was attached to the virtual machine, and was configured with a snapshot schedule to automatically create images of the disk at regular intervals. These images acted as backups and were used for deploying a copy of the database in production mode for testing or archival purposes. Because the disk was a separate resource, it was straightforward to change the virtual machine configuration to add or remove vCPUs or memory as needed without affecting the database. We also configured the database to serve as the GraphQL endpoint for API requests, via the Neo4j GraphQL plugin.

### Database model

Job nodes were annotated with properties describing the job inputs, command, Docker image^11^, network configuration, and machine type; as well as runtime statistics that are useful for assessing system health. Neo4j did not provide a formal method for defining database schema other than constraints that could be used to control how nodes were created. Therefore, we relied on the GraphQL schema to define object types, corresponding to Neo4j node labels, and the kinds of properties and relationship types that could be associated with nodes having that label.

Briefly, we used two relationship models; one to define provenance and the other to define the genomic functional domain (Fig. 2). The provenance relationship types include “GENERATED” and “WAS_USED_BY” while the functional domain includes “HAS_BIOLOGICAL_OME”, “HAS_SEQUENCING_READS”, “HAS_VARIANT_CALLS”, and “HAS_QC_DATA”. The relationship patterns provide different pathways for traversing the graph, based on query type. The schema is included as part of the source code for Trellis on GitHub.

#### Database triggers

Database triggers are used to determine when to run Trellis operations including bioinformatics jobs. We coded each database trigger as a Python class and stored them in a module separate from the db-triggers service code. Each class had four methods; “__init__”, “check_conditions”, “compose_message”, and “_create_query”. When database metadata for an updated node was passed to the db-triggers service, it used that metadata to call the “check_conditions” method for each database trigger (Supplementary Fig. 1). This method compared a predefined list of conditions against the values in the node metadata dictionary to check if they were met. If so, the metadata was then used to generate a database query using the “_create_query” method and published it back to the “db-query” service using the “compose_message” method. While the “check_conditions” method checked that the node metadata matched the conditions for running the job associated with the trigger, the database query checked that the context of the node was also appropriate for launching a job. In the case of bioinformatics jobs, the context may be appropriate when (1) all inputs required for the job have been registered in the database, and (2) a job fitting the same parameters has not already been requested or run. However, the database query may also include clauses to check for any other contextual situations in the database that must be met before running a job. We provided all database triggers in the source code on GitHub.

#### Using semaphore nodes to avoid duplicate jobs

Because the database is the only stateful component of Trellis, there is no mechanism other than database queries to orchestrate workflows. When a node was updated, its metadata would be transmitted to the db-triggers service. If it activated any triggers, the corresponding trigger queries would be sent back to the db-query service, where they were used to query the database (Supplemental Fig. 1 Panel 2). However, because messages are communicated between Trellis services using an asynchronous message broker, race conditions can arise when multiple nodes activate the same trigger. This situation could occur when a job took a set of the same types of objects as inputs. For instance, when a sample was sequenced using multiple read groups, unaligned bam objects would have to be generated for each read group, before inputting all of them to the GATK variant calling task. In such a case, each object in the set would activate the database trigger that checked whether all members of the set were registered in the database before triggering the job. Because of the lag time between when nodes were updated in the database and when their triggers were run, it was possible that the database state had changed when the query ran. For instance, when a node was added to the database, the conditions for activating the job may not have been met, but by the time the query ran, they were met. This could cause multiple events to trigger the same job and generate duplicates.

To reduce the incidence of duplicate jobs, we included a clause in each trigger query to create a semaphore node labelled “JobRequest” (Supplementary Fig. 10). The semaphore node indicated to every other subsequent database query that a job had already been requested, and they should not return any positive results that would create a duplicate job. This minimized the incidence of duplicate jobs, though we observed a slight increase in duplicate GATK jobs later in the time window, after changing the database configuration to increase page cache and heap size (Supplementary Fig. 9). We believe the change in heap size reduced the efficiency of database garbage collection, leading to an increase in duplicate job request nodes. We identified two putative causes for the duplicates: (1) a duplicate job request node was registered in the database or (2) a duplicate job request message was delivered to the job-launching service by the Google Pub/Sub message broker. Of 733 duplicate GATK jobs, we found duplicate job request nodes for 80% of them (Supplementary Fig. 11). Because Google Pub/Sub operates using an at-least-once delivery model, it is expected that messages will always be delivered at least once but may be delivered multiple times. To resolve this, we set up a monitoring service to label and deactivate them. This monitoring service successfully deactivated 95% of duplicate Fastq-to-ubam and 90% of duplicate GATK jobs (Supplementary Fig. 12).

In our future work, we plan to implement a few strategies to further reduce duplication. Based on our observation that duplicate jobs are associated with duplicate job request nodes, these nodes will be added to the database by merge operations and include a binary property indicating whether the database query created the node or matched an existing node. Only responses for queries that create nodes will launch downstream jobs. Second is to change the way that duplicate jobs are deactivated. Currently this is performed by making an API call directly to the Google Compute Engine API to deactivate the virtual machine; however, based on our experiences, it was not always successful. In the future, we plan on using the ddel utility packaged with Dsub to request deactivation via the same Google Cloud Life Sciences API that is used for virtual machine provisioning. Third, we will add a service to track transmission of messages between Trellis services in order to trace the lineage of internal operations responsible for launching each job. This should help identify the root-cause of issues including job duplications. Finally, we will investigate the feasibility of integrating Neo4j with the Apache Kafka message broker to send metadata describing all database updates to a message topic. Unlike Google Pub/Sub, Apache Kafka offers exactly-once message delivery, which should eliminate one source of job duplications, and also provides a mechanism for decoupling database triggers from database queries. These strategies should make Trellis more robust and performant.

#### Representing Cromwell workflows in the database

Trellis is designed as a system that triggers each step in a workflow generically, and the standard provence pattern is: (:Blob)-[:WAS_USED_BY]- > (:Job)-[:GENERATED]- > (:Blob). However, in the case where workflow is packaged, e.g. the GATK workflow used for variant calling in MVP was defined using Workflow Definition Language (WDL) for use with Cromwell, we modified Trellis database model to accept the packaged workflow as it was (Supplementary Fig. 14). Because the individual workflow steps were managed by Cromwell instead of Trellis and such information was not accessible, we added a “LED_TO” relationship to describe the lineage of jobs created by Cromwell as part of a predefined workflow. In this model, an initial node represented the workflow and each subsequent step branched off from it, e.g. (:CromwellWorkflow)-[:LED_TO]- > (:CromwellStep)-[:LED_TO]- > (:CromwellStep). Echo object output from a step directly related to the node representing the step. Because each step could involve multiple attempts executed on different virtual machines due to the parallel computing, each attempt was represented as a separate node and the attempts were linked together in order of their operation by “AFTER” relationships, e.g. (:CromwellStep)-[:LED_TO]- > (:CromwellStep)-[:GENERATED_ATTEMPT]- > (:CromwellAttempt)-[:AFTER]- > (:CromwellAttempt). One reason for this model was to exclude attempts from the “GENERATED”/”WAS_USED_BY”/”LED_TO” provenance chain to avoid cluttering query results aimed at retrieving data lineage, especially since steps such as haplotype calling included at least 50 attempts attributed to job sharding. Another reason was that metadata related to attempts was not that useful beyond knowing how many attempts were involved in a step, and chaining them all together made it easier to query. Although we recommend writing Trellis wrappers for every step in a workflow and letting Trellis manage the workflow orchestration in order to achieve the best performance, we also recognize the value of using well-established resources. Our amendment of the database model to accommodate Cromwell workflows proved the flexibility of the system.

### Service architecture

Services use the Cloud Pub/Sub message broker to communicate with each other. Each service has a dedicated Pub/Sub message topic from which it receives messages, and in turn communicates by sending messages to other topics. Trellis operations are “event-driven” because they are initiated by changes in the storage buckets that it monitors (Fig. 1). When a new data object is added to a Trellis-managed bucket, the storage service sends metadata about the object, or “blob”, to the “create-blob-node” service. This service will add domain-specific metadata (e.g. sample, cohort, chromosome) to the metadata dictionary and then publish it to the “db-query” service. The “db-query” service is responsible for communicating changes in the system to the database. It will create a new node in the database to represent the object, and then send the node metadata to the “db-triggers” service. This service is responsible for checking the node metadata against a set of database triggers. Because Trellis is an event-driven system, all operations are determined by metadata. If a particular trigger is activated, the query associated with that trigger will be run against the database, and the result sent to the topic specified in the trigger. For instance, an object of type “Bam”, containing sequencing reads data, can activate a trigger that will get the node metadata for that object and send it to the “launch-fastqc” service which will initiate a FastQC job that uses the Bam object as input. Once a service has initiated a job, the job metadata is passed to the “create-job-node” service which will parse and format it so it can also be added as a node in the database. And once the output of the job is added to a storage bucket, the cycle will begin again.

In addition, to handle the case where data has already been delivered to a cloud bucket, because there is no “event” associated with data already in storage, we created functions in the “Data Import” category to import static data and update the object’s metadata with an arbitrary key-value pair. We used the Cloud Scheduler service to create CRON jobs to initiate database triggers that would launch batches of particular jobs for samples that had not yet been analyzed.

#### Task management

Overall, we achieved a consistently high daily success rate when processing more than 81,000 genomes nonstop within a few months. Although in four short time periods we observed significant drops in success rates for Cromwell jobs running the GATK pipeline (Supplementary Fig. 5), analysis of the Cromwell logs files indicated that the root cause was temporary platform-side issues. The first drop occurred on May 5th and was traced back to a Google acknowledged bug that caused workflows using preemptible virtual machines to fail. On July 6th, 118 GATK jobs failed. Of these, 95% of job logs included HTTP response exception: 429, indicating too many server requests. Of the 218 jobs that failed on August 31st, 95% reported a storage exception with reading or writing data to cloud storage. And on September 25th, all of the failed jobs reported an HTTP response exception: 502 Bad Gateway. In each instance, the issues stopped occurring without changing any Trellis settings, and reflected transient platform issues.

#### Performance

Trellis relies on database triggers to mediate operations, and therefore database throughput is the primary factor limiting the number of jobs that can be run in parallel. Our implementation used the open-sourced Neo4j Community Edition, which was limited to using 4 vCPUs. Analysis of our operational model (Fig. 3c) revealed that the database could consistently run queries in under 350 ms at a rate of 25 queries per second, and queries were processed within 10 s of publication to the message broker. This performance remained consistent when the database size exceeded 93 million nodes and 98 million relationships, representing 81,000 MVP genomes. To maintain this throughput and avoid overloading the database, we capped the maximum number of instances of the database-querying at 20. This capacity could be expanded by using the paid-for enterprise edition of Neo4j, which would remove the vCPU cap and distribute the database across multiple machines. However, even operating under the constraints of the community edition of Neo4J, we found that the database proved sufficient to track our data and coordinate workflows.

### Job monitoring

In addition to the core architecture, we designed five functions in Trellis to monitor job states (Supplementary Table 5). Whenever a new job VM was created, the action was logged in Google’s Stackdriver monitoring system. We created custom alerts to send the metadata associated with these creation events to one of two functions: log-insert-trellis-instance for Dsub VMs or log-insert-cromwell-instance for Cromwell VMs. This included metadata describing the VM configuration, network, and creation time. Similarly, when a VM was deleted, because it had finished its job or failed, the same mechanism would send that metadata to the log-delete-instance function. These functions would parse the metadata and pass it to the db-query function so that it could be added to the job node. This allowed us to document the computing environment of each job as well as runtime. When a Dsub job node was updated with metadata indicating it had been deleted, it would trigger the check-dstat function. It used the Dstat command-line tool included with Dsub, to collect metadata describing whether the job succeeded, and any error messages. This information was also added to the job node in the database.

In addition to tracking job-specific metadata in the database, we also used a Monitoring dashboard to track the overall system health and performance of Trellis. We created several dashboard panels that each pull a subset of metadata from the Stackdriver monitoring logs, and generate visualizations summarizing all the metadata (Supplemental Fig. 15). We used the Monitoring system to generate alerts that were triggered whenever a summary metric exceeds a given threshold. These alerts were immediately delivered through our Slack messaging application so that an administrator could respond in time.

### Automated visualization of status and results

System metrics were quantified on a daily basis via a Jupyter notebook that used Neo4j database queries to collect summary metrics. These metrics were then uploaded to a BigQuery relational database, where they were processed and used by Google Datalab to generate automated visual reports (Supplemental Fig. 4). Our notebook code is included with the source materials on Github.

### Deployment of Trellis

We used Terraform and Google Cloud Build to automate as much as possible the deployment of Trellis resources. These “configuration-as-code” tools used configuration files to define how resources should be provisioned on the cloud. Using configuration files allows for uniform deployment of Trellis architecture across different cloud environments (e.g. development, test, and production). It also makes it easier to share the deployment configuration, so that other groups can reproduce our Trellis cloud environment. Though these methods are designed to ease the process of deploying Trellis, it is still a complex cloud system. We recommend that any potential user familiarize themselves with cloud platforms and networking before trying to use Trellis. Our deployment configurations as well as instructions are available via GitHub (https://github.com/StanfordBioinformatics/trellis-mvp-terraform).

### Security

In order to create a safe environment to run genomic data on the cloud, we designed to run Trellis as a closed system, i.e. without any connections outside of Google networks (Supplementary Fig. 3). We were able to do so because Trellis used an event-driven architecture, and most Trellis components communicated via Google services. Our serverless functions that ran all Trellis services communicated with each other via the Cloud Pub/Sub messaging service. Trellis maintained two persistent VMs, one for the database and one for a Jupyter analysis notebook that regularly generated summary statistics. The workflow described in an earlier section (“Genomics Workflow”) was run across thousands of ephemeral job VMs that ran in bursts to complete single tasks. All of these machines ran in a Virtual Private Cloud (VPC) in the us-west1 region of GCP. To minimize security risks, none of the machines were assigned external IP addresses. Only two functions connected to the database, db-query and db-query-index, and these used the Serverless VPC Connector service to avoid sending messages over the internet.

Even though Trellis is event-driven, there are cases where the database or analysis notebook needs to be accessed to do maintenance, perform updates, or investigate issues. To accommodate this, we set up a bastion node in a separate project that users can connect to. This node was protected by firewall rules which only allowed access on port 22 (SSH protocol) by two designated IP addresses, corresponding to two physical ports at the work stations of MVP service core team members in a secure building belonging to the Stanford School of Medicine. To account for the possibility that system administrators may need to work remotely for extended periods, port 22 access has also been granted to the IP address corresponding to one of the login nodes of the high-performance computing cluster maintained by the Stanford School of Medicine. The Trellis VPC firewall only allows SSH access from the first bastion host. However, once a user accessed the bastion, they could then use their Google credentials to access a second bastion host within the Trellis VPC network to expand their functionality. We only activated these bastion nodes during maintenance periods.

With respect to the overall computing environment, Google Cloud is compliant with HIPAA, FEDRAMP, and FISMA-moderate Authorization, and data on Google Cloud is encrypted at all times. In addition, we accessed Google Cloud via the Stanford University gateway and therefore are protected by Stanford University’s Business Association Agreement for GCP. The way we managed Trellis as a closed system on GCP ensured that all computational tasks related to MVP genome processing and QC were carried out in a highly secure manner.

### GitHub sites

https://github.com/StanfordBioinformatics/trellis-mvp-gatk.

https://github.com/StanfordBioinformatics/trellis-mvp-functions.

https://github.com/StanfordBioinformatics/trellis-mvp-terraform.

https://github.com/StanfordBioinformatics/trellis-mvp-api.

https://github.com/StanfordBioinformatics/trellis-mvp-analysis.

## Supplementary Information

Supplementary Information.
