# Supplementary material for: Trellis for efficient data and task management in the VA Million Veteran Program
Source: Sci Rep. 2021 Dec 1;11:23229. doi: 10.1038/s41598-021-02569-5 (PMC8636485; doi:10.1038/s41598-021-02569-5)
Supplement: Supplementary file 1 — Supplementary Information. [file 41598_2021_2569_MOESM1_ESM.pdf]

Supplemental Material

Trellis for Efficient Data and Task Management in the VA Million Veteran Program

Paul Billing Ross[1,3], Jina Song[1,3], Philip S. Tsao[2,3,*], Cuiping Pan[3,*]

[1] Stanford Center for Genomics and Personalized Medicine, Stanford University, CA

[2] Department of Medicine, Stanford University, CA

[3] Palo Alto Epidemiology Research and Information Center for Genomics, VA Palo Alto, CA

*Correspondence to: pstao@stanford.edu, cuiping@stanford.edu

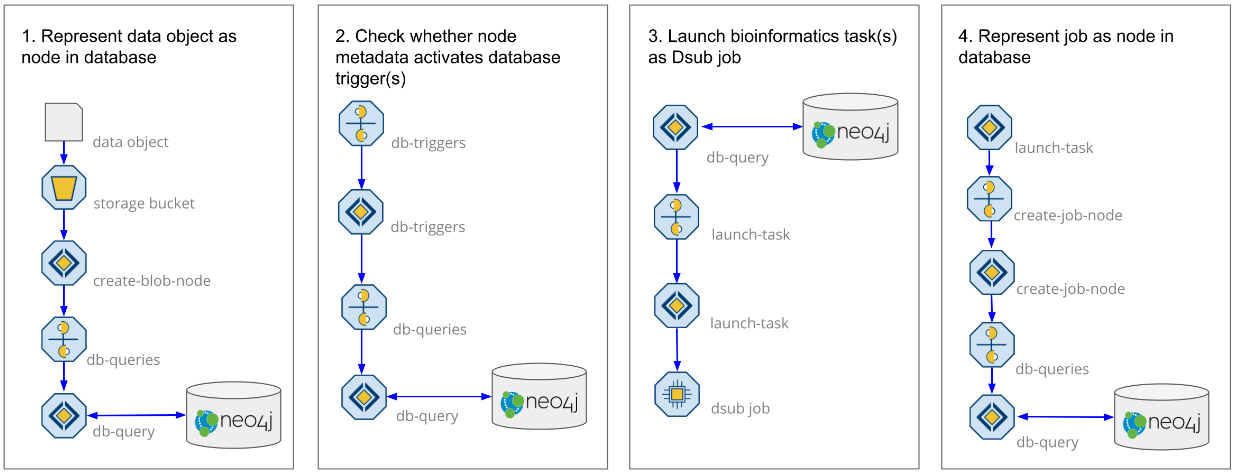

Supplementary Figure 1. Four core functionalities of Trellis. When a new data object is added to a Trellis-managed bucket, the storage service sends metadata about the object, or "blob", to the "create-blob-node" service. This service will add domain-specific metadata (e.g. sample, cohort, chromosome) to the metadata dictionary and then publish it to the "db-query" service. The "db-query" service is responsible for communicating changes in the system to the database. It will create a new node in the database to represent the object, and then send the node metadata to the "db-triggers" service. This

service is responsible for checking the node metadata against a set of database triggers that are used to launch bioinformatics jobs and trigger Trellis operations. Each trigger is associated with a database query that will be run if the trigger is activated. Upon activation, the query is sent back to the "db-query" service where it will be run against the database. The metadata results of the query are then sent to the appropriate service to launch the corresponding job.

(a)

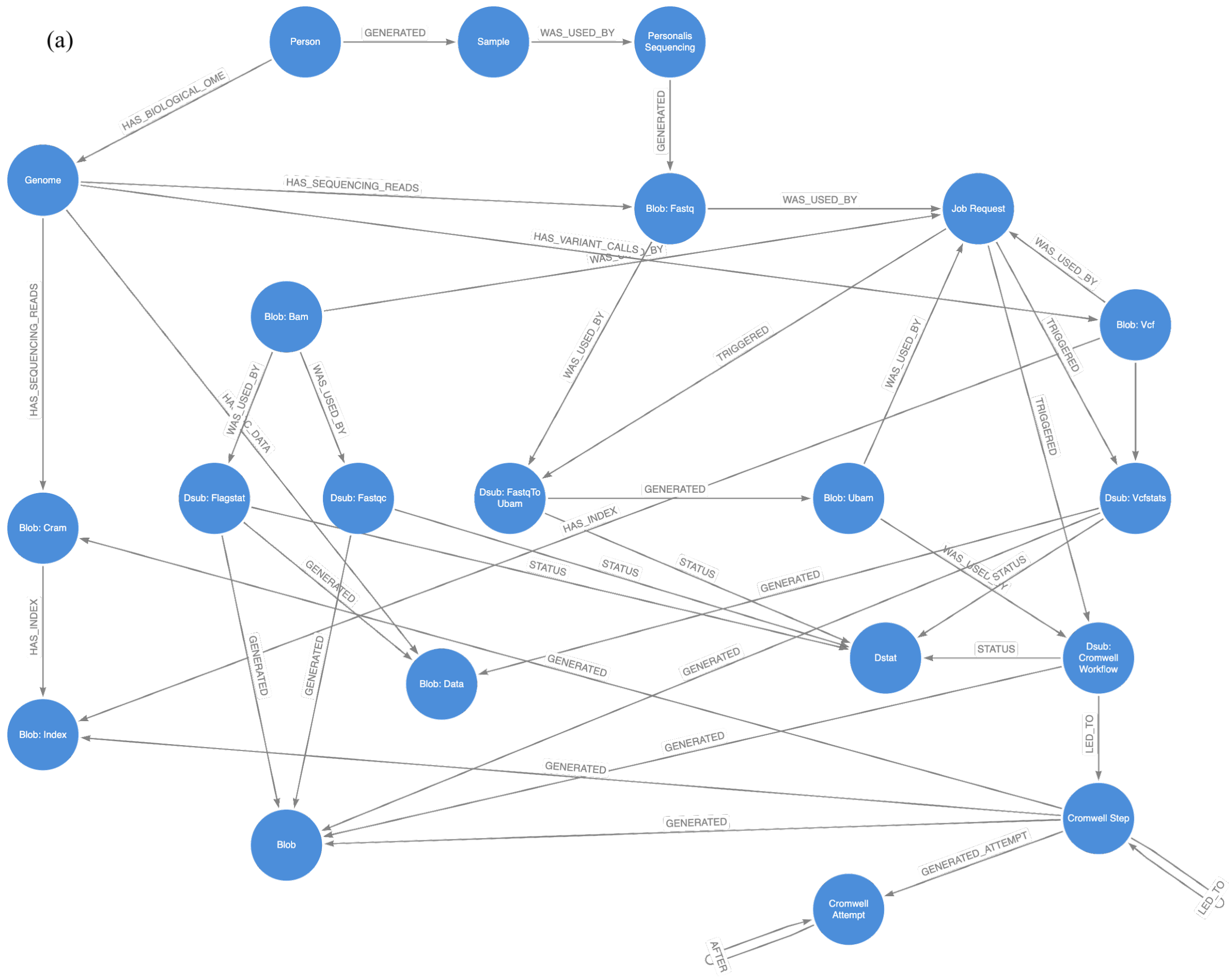

Person
GENERATED
Sample
WAS_USED_BY
Personalis Sequencing
HAS_BIOLOGICAL_OME
Genome
HAS_SEQUENCING_READS
GENERATED
Blob: Fastq
WAS_USED_BY
Job Request
HAS_VARIANT_CALLS
WAS_USED_BY
Blob: Vcf
Blob: Bam
TRIGGERED
HAS_QC_DATA
Dsub: Flagstat
Dsub: Fastqc
Dsub: FastqTo Ubam
Blob: Ubam
Dsub: Vcfstats
Blob: Cram
HAS_INDEX
STATUS
Dstat
Dsub: Cromwell Workflow
Blob: Data
Blob: Index
LED_TO
Cromwell Step
Blob
GENERATED_ATTEMPT
Cromwell Attempt
AFTER

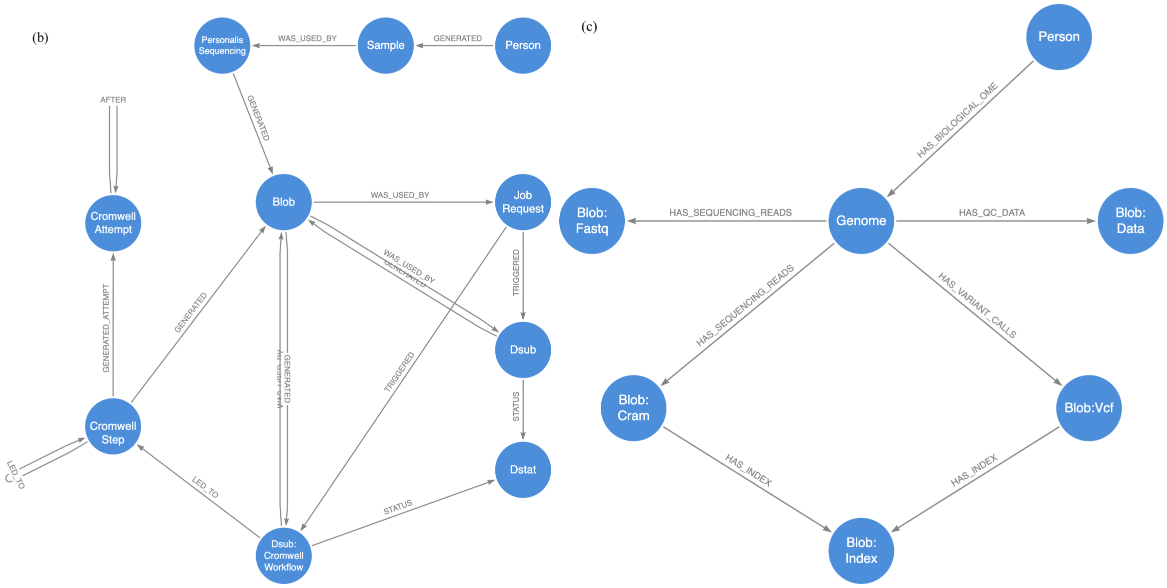

Supplementary Figure 2. Graph representation of the Trellis data model. Panel (a) includes the entire graph model while panel (b) describes the portion of the data model connected by provenance relationships and (c) describes the part connected by relationships in the genome functional domain.

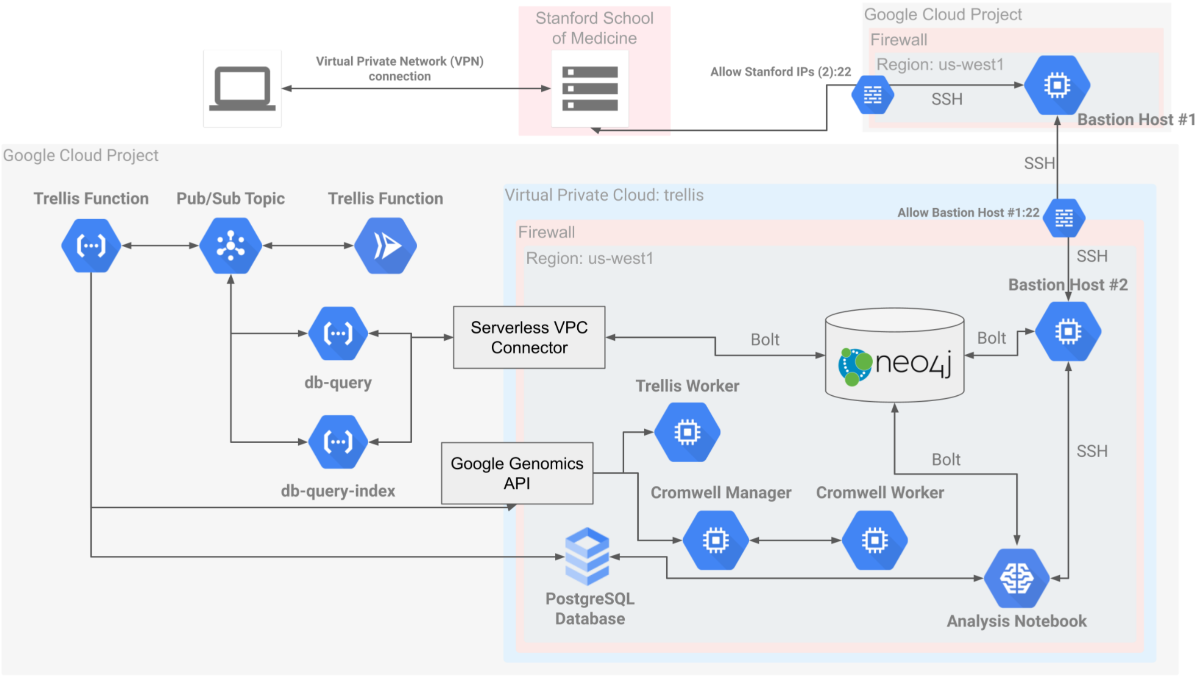

Supplementary Figure 3. Deploying the Trellis model on GCP in a secure manner. Network diagram describing the patterns of information flow between individual Trellis resources and between users and Trellis resources. Resources have been organized to minimize external access points to Trellis resources.

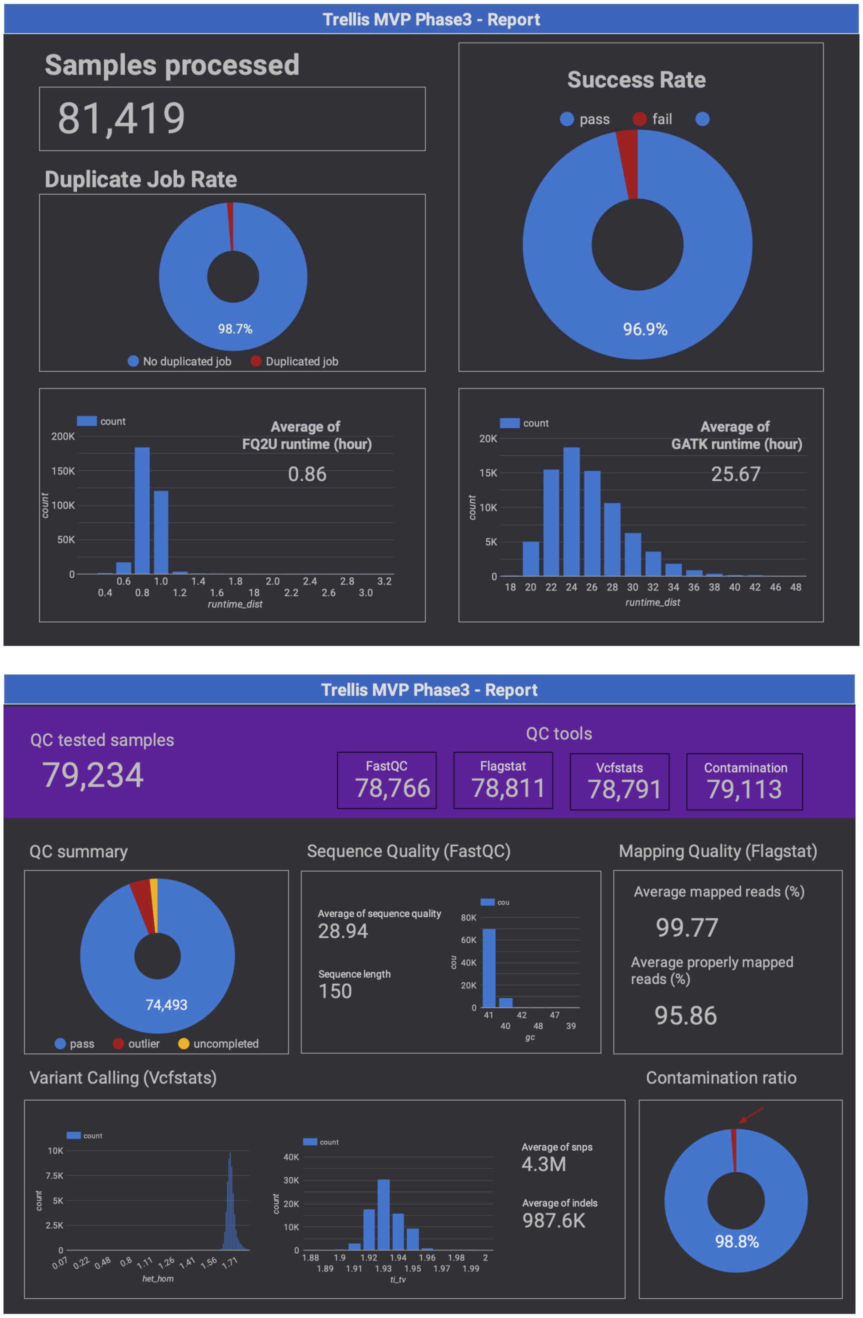

Supplementary Figure 4. An example of the daily report of data processing status and summary statistics of the genomes.

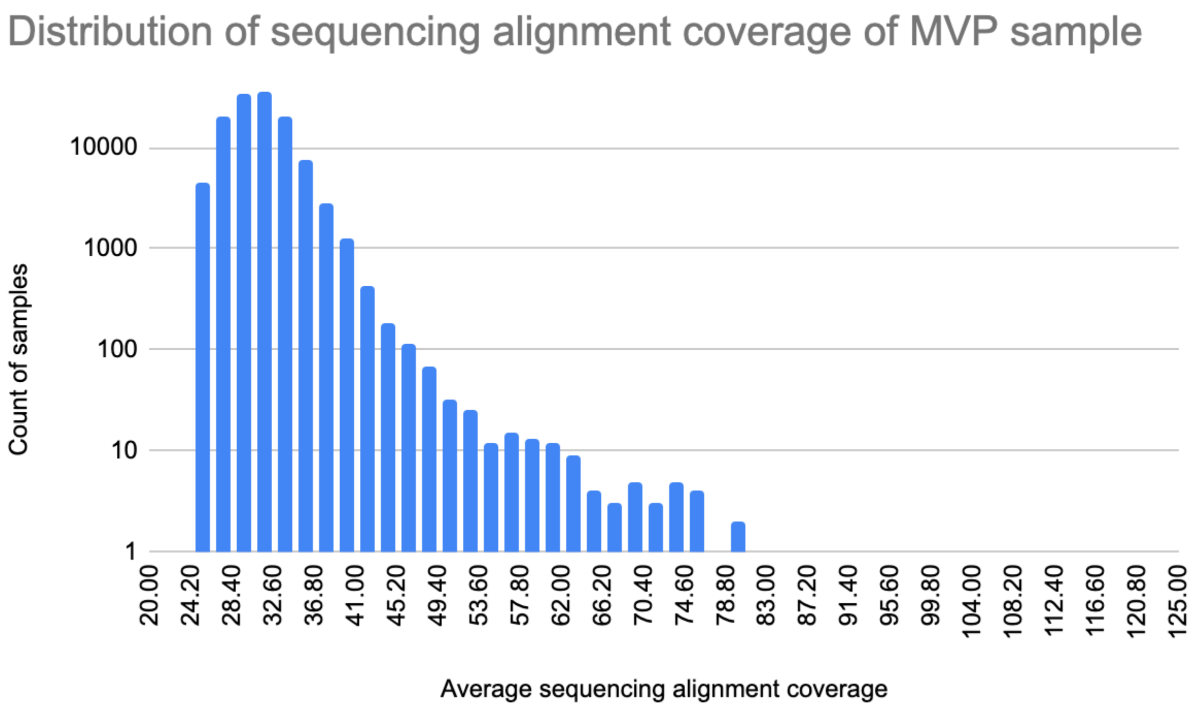

Supplementary Figure 5. Distribution of average sequencing alignment coverage for Million Veteran program whole-genome sequenced samples.

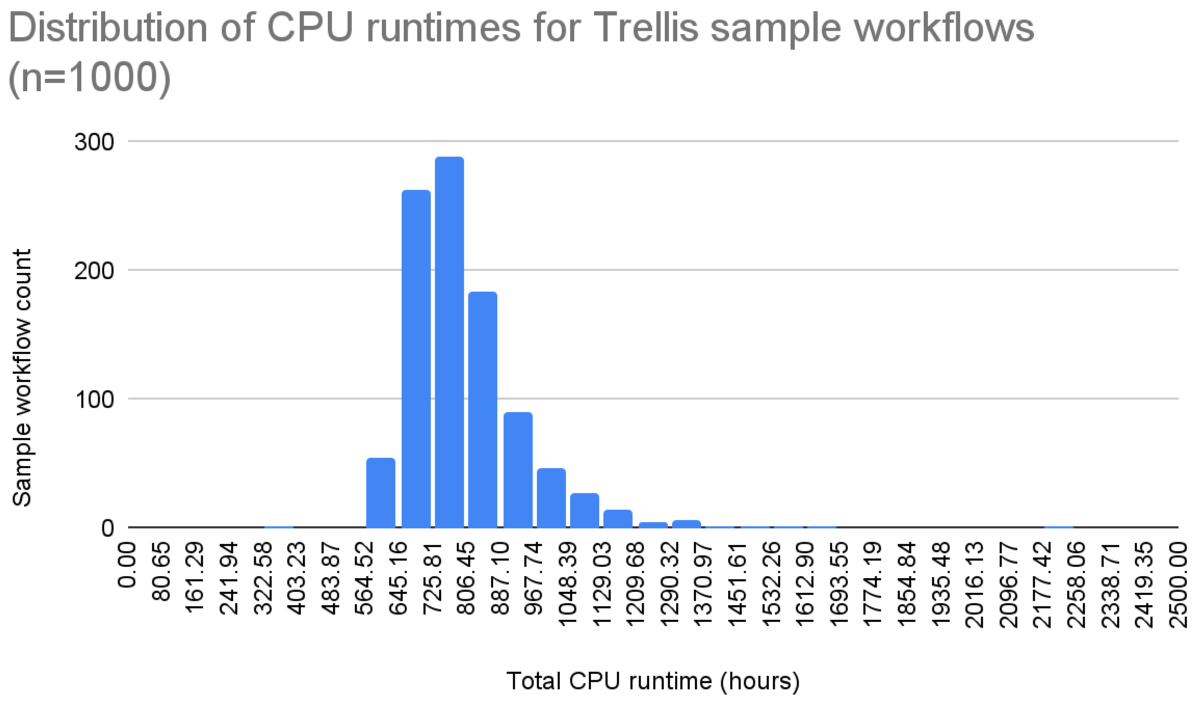

Supplementary Figure 6. Distribution of total CPU runtimes for running an end-to-end variant calling and quality control-workflow on a single whole-genome sequencing sample. CPU runtime was calculated by multiplying the number of CPUs used for each step in the workflow by the runtime of the task.

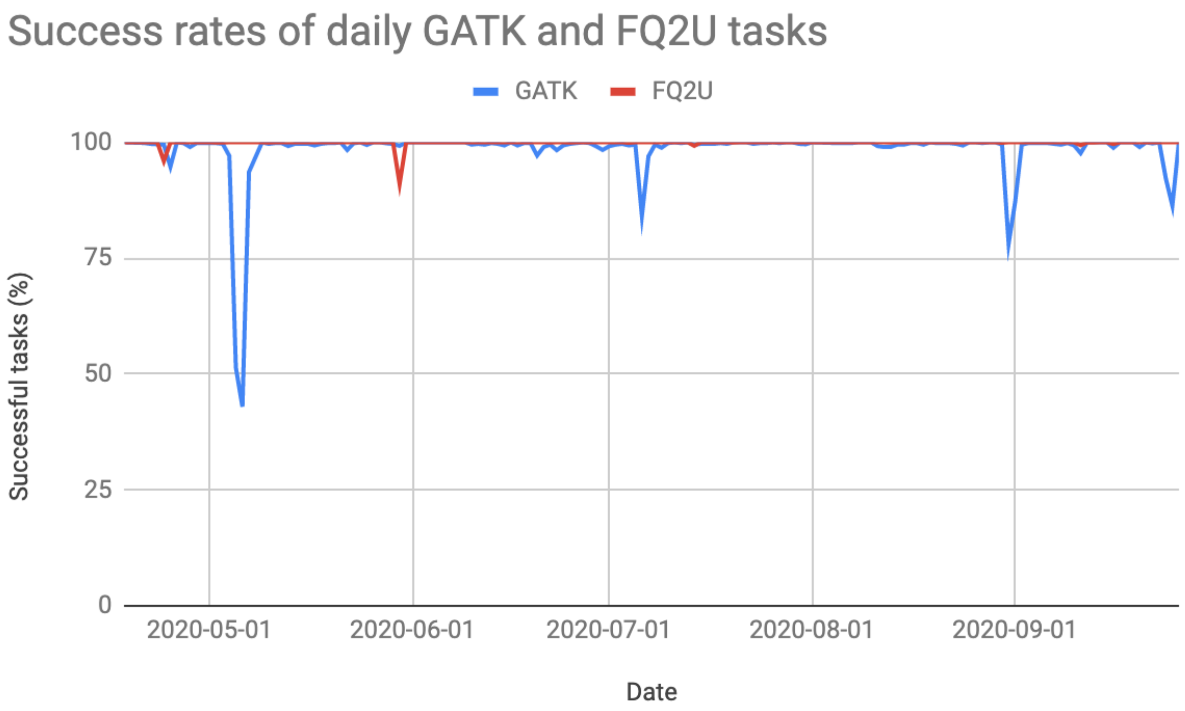

Supplementary Figure 7. Daily success rates of the two primary Trellis jobs responsible for variant calling; the Cromwell GATK pipeline and fastq-to-ubam conversion. The four precipitous decreases in Cromwell GATK success rates were all associated with temporary GCP platform-side issues, including: May 5th - A Google API error that affected the preemptible VMs used by Cromwell., July 6th - HTTP response exception, August 31st - storage I/O exception, and September 25th - HTTP response exception.

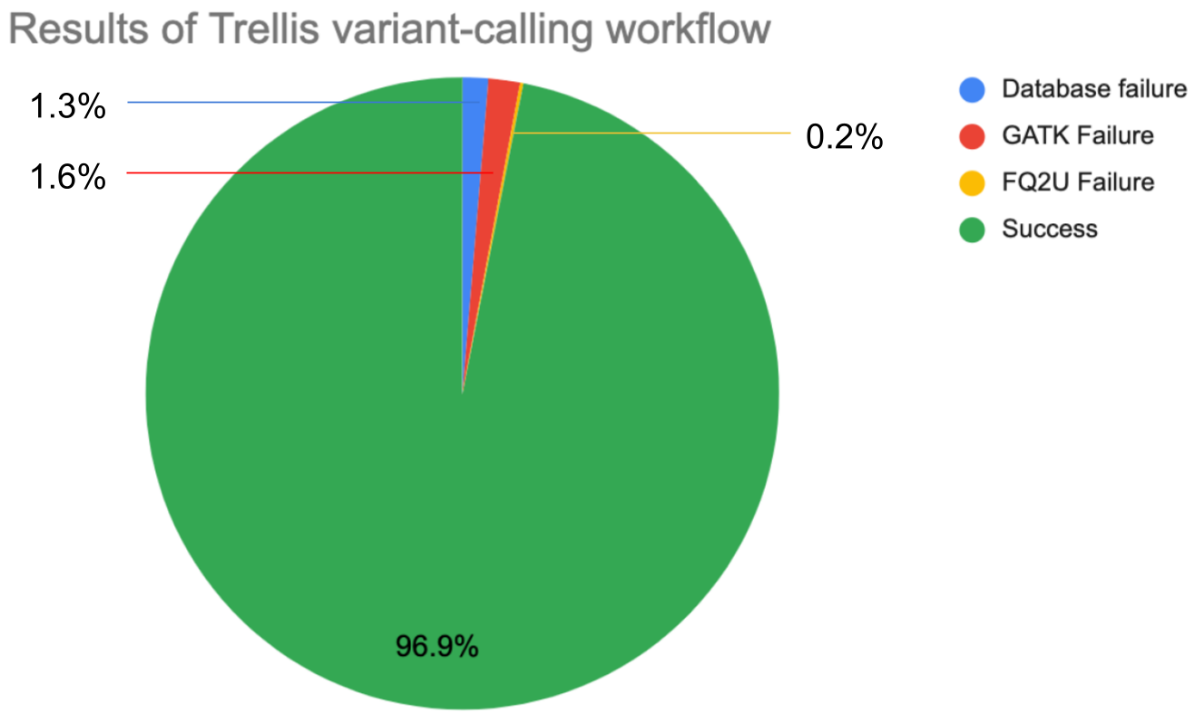

Supplementary Figure 8. Results of Trellis variant calling workflow for 81,791 genomes. The workflow involves running fastq-to-ubam (FQ2U) on all Fastq read groups to generate Ubams which are used as input to the GATK germline variant calling workflow which is run using Cromwell (GATK). Workflows were successful in 96.9% of cases and failed to complete in 3.1% of cases. Database failures accounted for 43% (1.3% of total) of workflow failures. In these instances, a database discrepancy or networking issue resulted in workflows not completing. Failures due to Cromwell errors while running the GATK workflow led to 52% of failure cases (1.6%), while Dsub failures while running fastq-to-ubam jobs resulted in the final 5% of failures (0.2%).

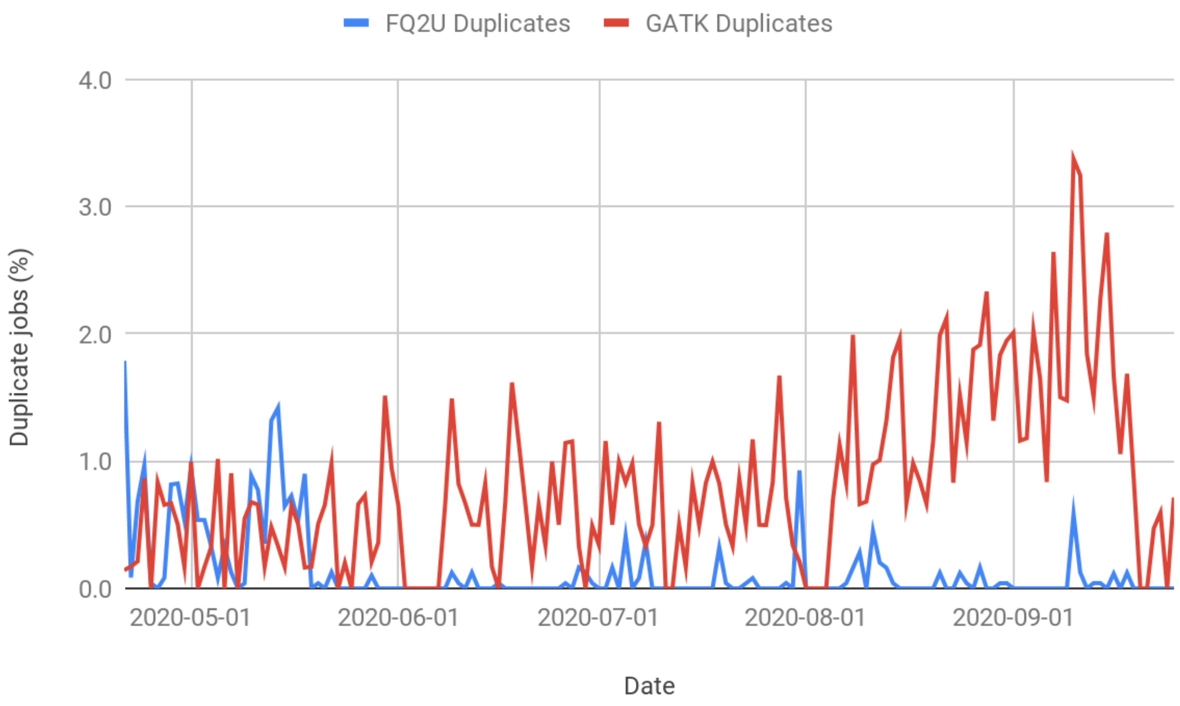

Supplementary Figure 9. Rate of duplicate jobs launched for the two primary variant calling tasks managed by Trellis: the preprocessing step - Fastq to Ubam conversion (FQ2U) and the actual processing - the GATK workflow.

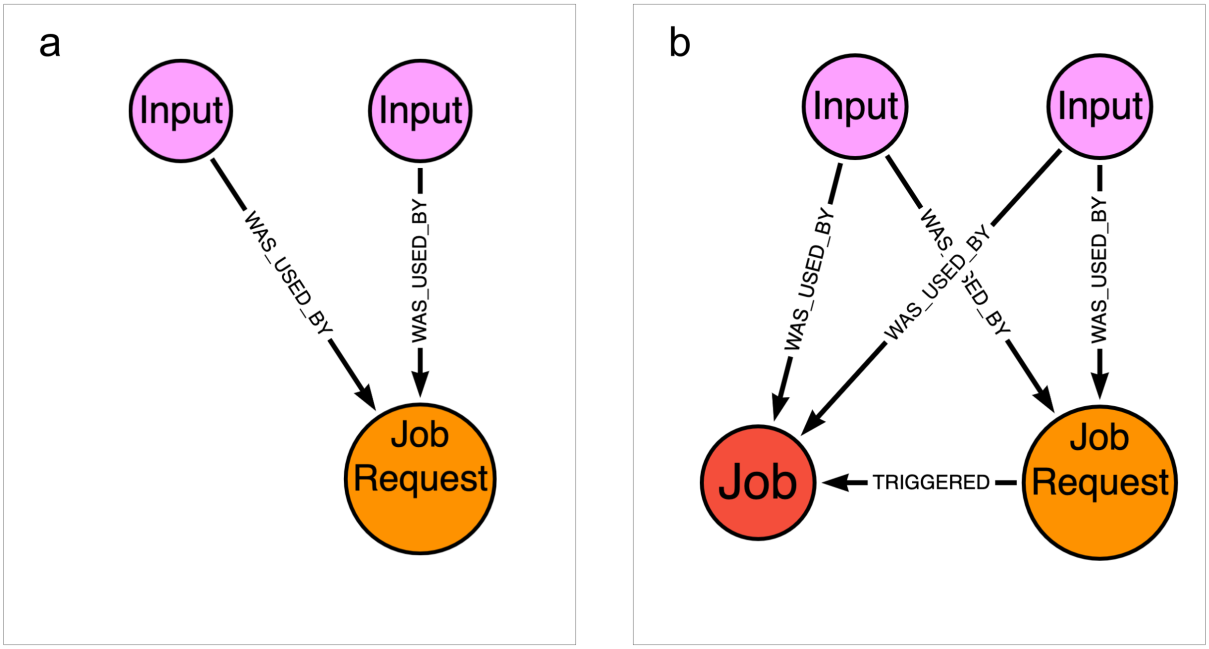

Supplementary Figure 10. Illustration of how semaphore nodes are used to reduce duplicate jobs. When a database triggered query finds that the conditions are appropriate for launching a job, a semaphore node labelled "JobRequest" is created to block any similar queries from triggering a duplicate job (Panel A). This node is created by the query so that the job request is immediately registered in the database. This alleviates the problem of duplicate jobs being launched in the interim period between when the trigger query completes and when the job node is added to the database (Panel B).

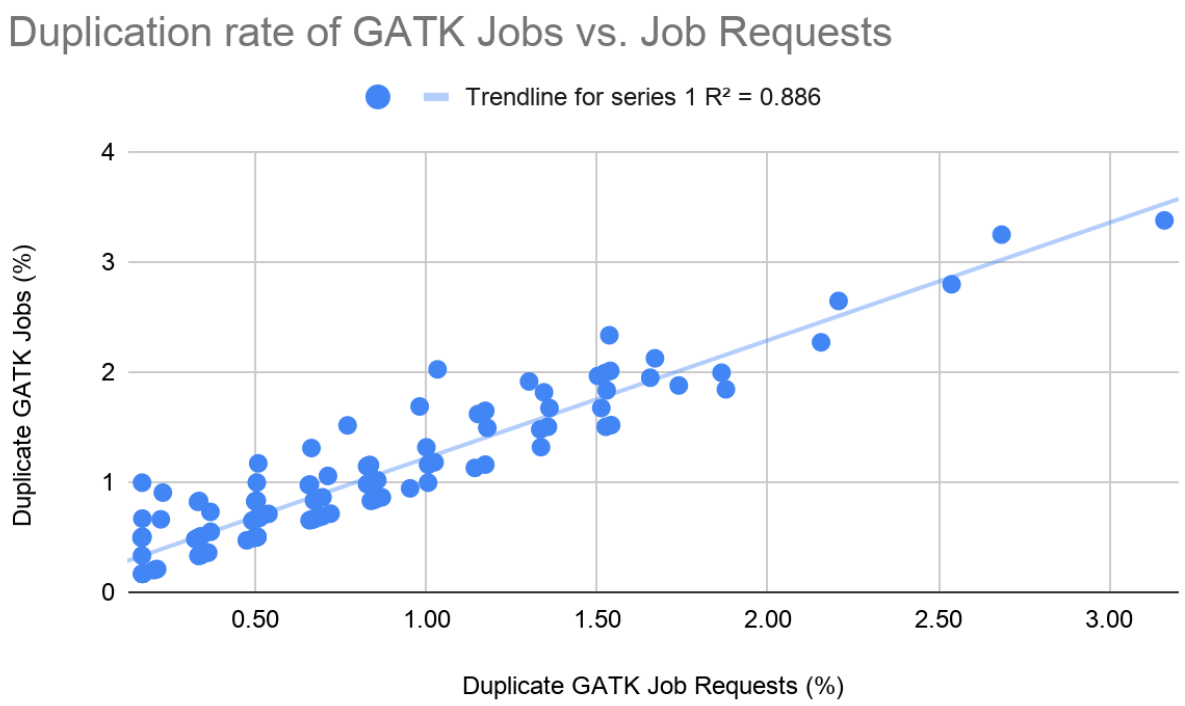

Supplementary Figure 11. Comparison between rate of duplicate GATK jobs per day and rate of duplicate GATK job requests. The high positive correlation (R^2=0.88) indicates that duplicate job requests registered in the Neo4j database were the primary drivers of duplicate GATK jobs being launched.

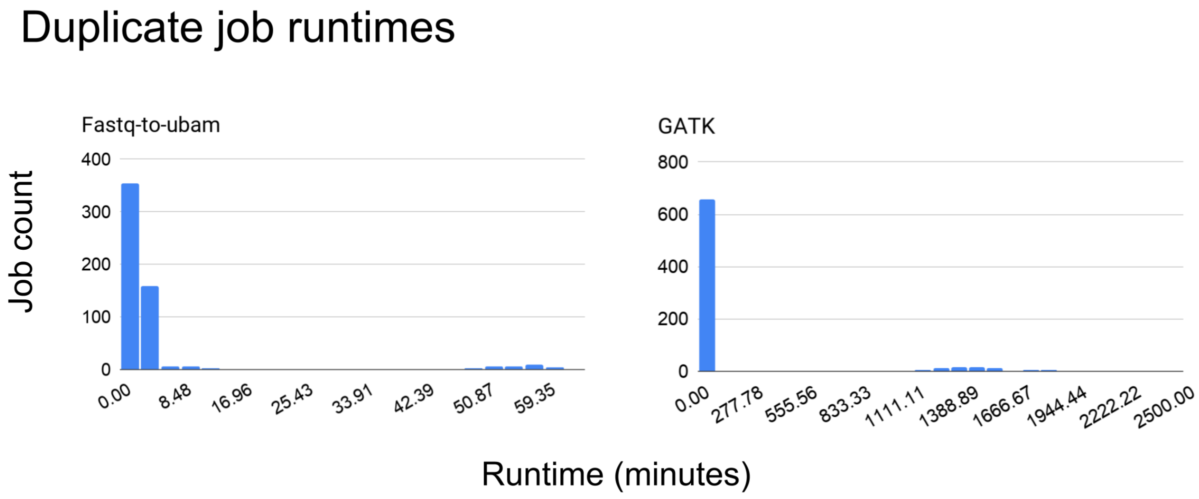

Supplementary Figure 12. Distribution of runtimes of duplicate jobs. The bimodal distribution shows that 95% of duplicate Fastq-to-ubam jobs and 90% of duplicate GATK variant-calling jobs were stopped before reaching completion. The average runtime for jobs stopped by Trellis was 2.3 minutes for fastq-to-ubam and 2.6 minutes for GATK jobs. Jobs that were not successfully stopped ran to completion.

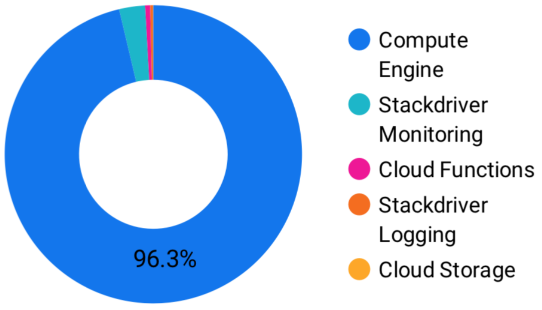

Supplementary Figure 13. Cost distribution among various computational functions in the Trellis-managed workflow of variant calling and quality assessments.

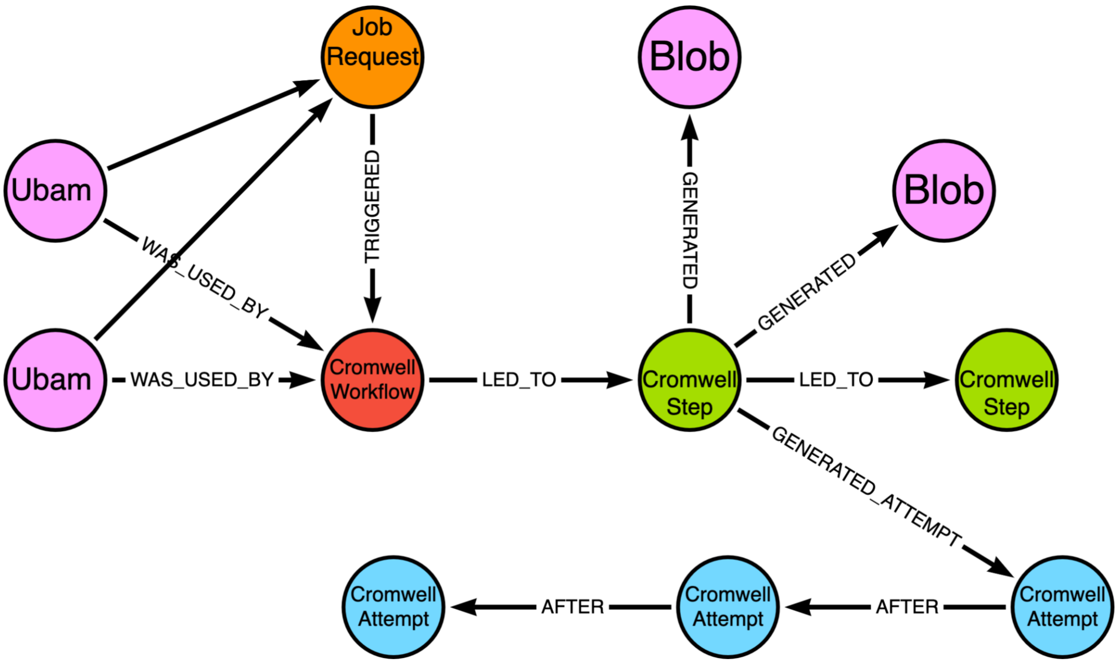

Supplementary Figure 14. Depiction of how the Trellis database model was modified to accommodate self-contained Cromwell workflows. The "LED_TO" relationship type was added to describe the provenance of individual Cromwell steps within a Cromwell workflow. The "CromwellAttempt" node labels were used to represent each instance of a virtual machine that was used to run a Cromwell step. Because the number of virtual machines used for each step varied, the "GENERATED_ATTEMPT"/"AFTER" relationships were used to link attempts together in the interest of monitoring resource usage at each step.

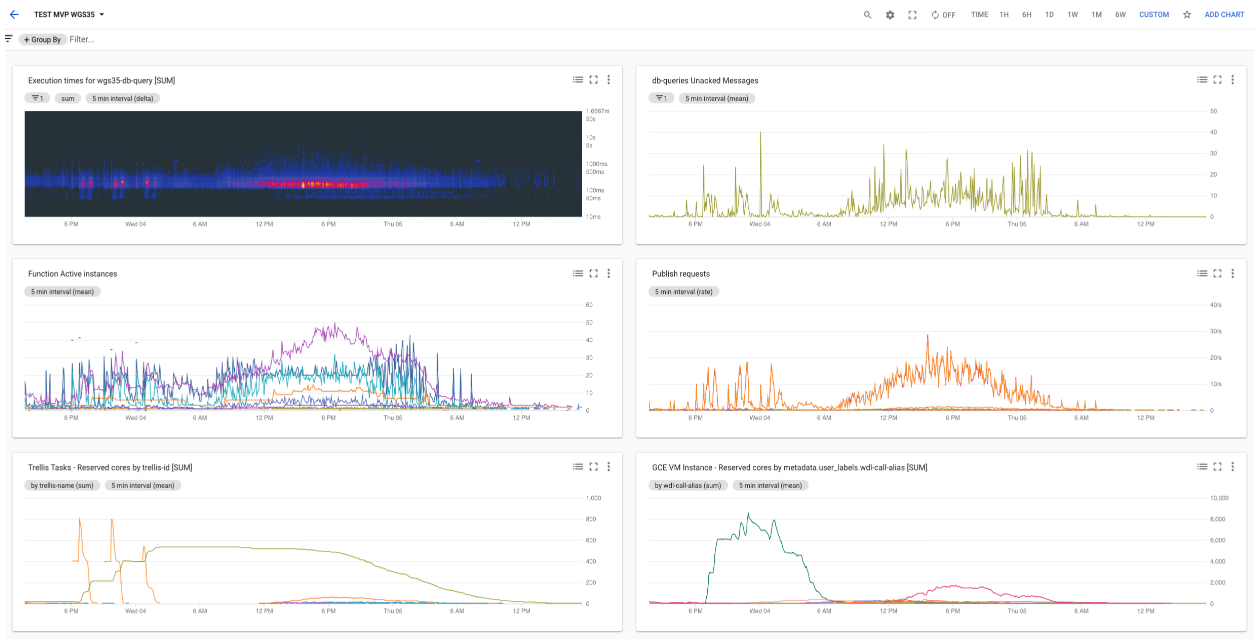

Supplemental Figure 15. Stackdriver dashboard presents performance of the Trellis system. Shown in this example figure are the database query executive times, published number of messages, the speed at which Pub/Sub messages were processed, active functions at short time intervals, cumulative number of VMs for the workflow, etc.

Supplementary Table 1. Quality control steps implemented in Trellis for whole genome sequencing data.

Analysis	Tool	Input	Results
**Sequencing Reads**	FastQC 0.11.4	bam	● Base sequence quality<br>● Read quality<br>● GC contents
**Read Alignment**	Samtools 0.1.19 (flagstat)	bam	● Mapped reads<br>● Properly mapped reads
	GATK (verifybamid)	bam	● DNA contamination rate
**Variant Calling**	RTG Tools 3.7.1 (vcfstats)	gvcf	● Counts of SNV, Indels,<br>● SNV Ti/Tv<br>● SNV Het/Hom

Supplementary Table 2. Breakdown of the cost of running variant calling and QC applications on a single MVP WGS sample. Costs are organized by GCP stock keeping units (SKU) and product. Costs are based on processing 288 MVP samples and units are in USD. Only 12 of the 17 SKUs that generated costs greater than $0.01 are listed.

	SKU	Product	Subtotal per sample ▾
1.	Preemptible Custom Instance Core	Compute Engine	2.23
2.	Custom Instance Core	Compute Engine	1.7
3.	Custom Instance Ram	Compute Engine	1.3
4.	Storage PD Capacity	Compute Engine	0.75
5.	Preemptible Custom Instance Ram	Compute Engine	0.72
6.	N1 Predefined Instance Core	Compute Engine	0.57
7.	N1 Predefined Instance Ram	Compute Engine	0.39
8.	Monitoring Metric Volume	Stackdriver Monitoring	0.22
9.	Cloud Function CPU Time	Cloud Functions	0.03
10.	Log Volume	Stackdriver Logging	0.03
11.	Cloud Function Invocations	Cloud Functions	0.01
12.	Static Ip Charge	Compute Engine	0.01
		**Grand total**	**7.97**

Supplementary Table 3. Monthly per-sample cost of keeping essential whole-genome sequencing data on Google Cloud Storage. Costs calculated using Google Cloud pricing (https://cloud.google.com/storage/pricing).

**Object Type**	**Mean Size (GB)**	**Storage Class**	**Mean Monthly Storage Cost ($)**
Fastq	49	Coldline	0.196
Cram	9.6	Standard	0.192
gVCF	6.2	Standard	0.124
**Combined**	**64.8**	-	**0.512**

Supplementary Table 4. Docker images used by Trellis to perform germline variant calling and quality control.

Docker image	Description	Purpose
broadinstitute/cromwell:53	Cromwell workflow engine	Running GATK variant-calling workflow
broadinstitute/gatk:4.1.0.0	Toolkit for genomic data	Running GATK tasks
realtimegenomics/rtg-tools:3.7.1	Toolkit for use with VCF data	Running Vcfstats (QC)
biocontainers/samtools:v1.9-4-deb_cv1	Toolkit for use with sequencing data	Running Flagstat (QC)
stanfordbioinformatics/text-to-table:0.2.1	Tool for converting structured text data to table format	Converting results of QC tools to table format
biocontainers/fastqc:v0.11.5_cv4	Tool for generating QC metrics from sequencing data	Running FastQC (QC)
neo4j:3.5.14	Open-source Neo4j graph database	Running the Trellis graph database

Supplementary Table 5. Configuration of the serverless functions in Cloud Run/Functions in Trellis for processing 100,000 genomes in the MVP. The serverless functions have been grouped into functional categories. Core functions are essential to Trellis operations. Monitoring functions are used to track metadata describing virtual machine runtimes and failure rates. Functions in the “ Data Import” category are used to import static data, i.e. those that have already been delivered to a cloud bucket, into the database. Because there is no “event” associated with data already in storage, these functions create an event for each object to be imported, by updating the object’s metadata with an arbitrary key-value pair.

**Function Name**	**Functional Category**	**Managed Service**	**Memory (MB)**	**Max Instances**	**Language Runtime**
check-dstat	Job monitoring	Cloud Run	256	NA	NA
check-triggers	Core	Cloud Functions	256	20	Python 3.7
create-blob-node	Core	Cloud Functions	128	100	Python 3.7
create-job-node	Core	Cloud Functions	128	100	Python 3.7
db-query	Core	Cloud Functions	256	20	Python 3.7
db-query-index	Data import	Cloud Functions	128	10	Python 3.7
kill-job	Job monitoring	Cloud Functions	128	40	Python 3.7
launch-bam-fastqc	Job launcher	Cloud Functions	128	20	Python 3.7
launch-fastq-to-ubam	Job launcher	Cloud Functions	128	25	Python 3.7
launch-flagstat	Job launcher	Cloud Functions	128	20	Python 3.7
launch-gatk-5-dollar	Job launcher	Cloud Functions	128	10	Python 3.7
launch-text-to-table	Job launcher	Cloud Functions	128	20	Python 3.7
launch-vcfstats	Job launcher	Cloud Functions	128	20	Python 3.7
list-bucket-page	Data import	Cloud Functions	128	100	Python 3.7
log-delete-instance	Job monitoring	Cloud Functions	128	100	Python 3.7
log-insert-cromwell-instance	Job monitoring	Cloud Functions	128	100	Python 3.7

log-insert-trellis-instance	Job monitoring	Cloud Functions	128	100	Python 3.7
match-blob-patterns	Data import	Cloud Functions	128	100	Python 3.7
postgres-insert-data	ETL	Cloud Functions	256	5	Python 3.7
update-metadata	Data import	Cloud Functions	128	200	Python 3.7
blob-update-storage-class	Object management	Cloud Functions	128	200	Python 3.7
register-blob-deleted	Object management	Cloud Functions	128	100	Python 3.7
register-sample-snvqa-status	Object management	Cloud Functions	128	100	Python 3.7

Supplementary Table 6. Results of 332,392 individual Fastq-to-ubam jobs run for as part of the germline variant calling workflows for 81,184 samples. Descriptions of failures were inferred from the status messages output from the Dsub task manager.

Result	Description	Task Count	Task Percentage
Success	Success	332131	99.921
Failure	Timeout while pulling Docker image	237	0.071
Failure	Uncategorized	8	0.002
Failure	Hard disk space exceeded	5	0.002
Failure	Operation walltime exceeded	3	0.001
Failure	Data localization error	8	0.002

Supplementary Table 7. Results of individual Cromwell GATK germline variant calling tasks associated with 81,184 sample workflows. Descriptions of failures were inferred from the status messages output from Cromwell. The majority of failures are considered uncategorized because we have not yet implemented a method of extracting the error messages from the Cromwell log files and adding them to the Neo4j database. For 517 samples, this task was never started because of upstream failures.

Result	Description	Task Count	Task Percentage
Success	Success	79335	98.35
Failure	Operation walltime exceeded	82	0.10
Failure	Timeout pulling Docker image	35	0.04
Failure	Uncategorized	1215	1.51

Supplementary List 1:
https://github.com/StanfordBioinformatics/trellis-mvp-functions/blob/master/docs/trellis-bento-data-model.yaml

Supplementary List 2:
https://github.com/StanfordBioinformatics/trellis-mvp-functions/blob/master/docs/trellis-job-machine-types.csv

Supplementary List 3: VA Million Veteran Program: Core Acknowledgement for Publications (Updated December 10, 2020)

MVP Executive Committee

- Co-Chair: J. Michael Gaziano, M.D., M.P.H.
VA Boston Healthcare System, 150 S. Huntington Avenue, Boston, MA 02130
- Co-Chair: Sumitra Muralidhar, Ph.D.
US Department of Veterans Affairs, 810 Vermont Avenue NW, Washington, DC 20420
- Rachel Ramoni, D.M.D., Sc.D., Chief VA Research and Development Officer
US Department of Veterans Affairs, 810 Vermont Avenue NW, Washington, DC 20420
- Jean Beckham, Ph.D.
Durham VA Medical Center, 508 Fulton Street, Durham, NC 27705
- Kyong-Mi Chang, M.D.
Philadelphia VA Medical Center, 3900 Woodland Avenue, Philadelphia, PA 19104
- Christopher J. O'Donnell, M.D., M.P.H.
VA Boston Healthcare System, 150 S. Huntington Avenue, Boston, MA 02130
- Philip S. Tsao, Ph.D.
VA Palo Alto Health Care System, 3801 Miranda Avenue, Palo Alto, CA 94304
- James Breeling, M.D., Ex-Officio
US Department of Veterans Affairs, 810 Vermont Avenue NW, Washington, DC 20420
- Grant Huang, Ph.D., Ex-Officio
US Department of Veterans Affairs, 810 Vermont Avenue NW, Washington, DC 20420
- Juan P. Casas, M.D., Ph.D., Ex-Officio
VA Boston Healthcare System, 150 S. Huntington Avenue, Boston, MA 02130

MVP Program Office

- Sumitra Muralidhar, Ph.D.
US Department of Veterans Affairs, 810 Vermont Avenue NW, Washington, DC 20420
- Jennifer Moser, Ph.D.
US Department of Veterans Affairs, 810 Vermont Avenue NW, Washington, DC 20420

MVP Recruitment/Enrollment

- Recruitment/Enrollment Director/Deputy Director, Boston – Stacey B. Whitbourne, Ph.D.; Jessica V. Brewer, M.P.H.
  VA Boston Healthcare System, 150 S. Huntington Avenue, Boston, MA 02130
- MVP Coordinating Centers
  - Clinical Epidemiology Research Center (CERC), West Haven – Mihaela Aslan, Ph.D.
    West Haven VA Medical Center, 950 Campbell Avenue, West Haven, CT 06516
  - Cooperative Studies Program Clinical Research Pharmacy Coordinating Center, Albuquerque – Todd Connor, Pharm.D.; Dean P. Argyres, B.S., M.S.
    New Mexico VA Health Care System, 1501 San Pedro Drive SE, Albuquerque, NM 87108
  - Genomics Coordinating Center, Palo Alto – Philip S. Tsao, Ph.D.
    VA Palo Alto Health Care System, 3801 Miranda Avenue, Palo Alto, CA 94304
  - MVP Boston Coordinating Center, Boston - J. Michael Gaziano, M.D., M.P.H.
    VA Boston Healthcare System, 150 S. Huntington Avenue, Boston, MA 02130
  - MVP Information Center, Canandaigua – Brady Stephens, M.S.
    Canandaigua VA Medical Center, 400 Fort Hill Avenue, Canandaigua, NY 14424
- VA Central Biorepository, Boston – Mary T. Brophy M.D., M.P.H.; Donald E. Humphries, Ph.D.; Luis E. Selva, Ph.D.
  VA Boston Healthcare System, 150 S. Huntington Avenue, Boston, MA 02130
- MVP Informatics, Boston – Nhan Do, M.D.; Shahpoor (Alex) Shayan, M.S.
  VA Boston Healthcare System, 150 S. Huntington Avenue, Boston, MA 02130
- MVP Data Operations/Analytics, Boston – Kelly Cho, M.P.H., Ph.D.
  VA Boston Healthcare System, 150 S. Huntington Avenue, Boston, MA 02130
- Director of Regulatory Affairs – Lori Churby, B.S.
  VA Palo Alto Health Care System, 3801 Miranda Avenue, Palo Alto, CA 94304

MVP Science

- Science Operations – Christopher J. O'Donnell, M.D., M.P.H.
  VA Boston Healthcare System, 150 S. Huntington Avenue, Boston, MA 02130
- Genomics Core – Christopher J. O'Donnell, M.D., M.P.H.; Saiju Pyarajan Ph.D.
  VA Boston Healthcare System, 150 S. Huntington Avenue, Boston, MA 02130
  Philip S. Tsao, Ph.D.
  VA Palo Alto Health Care System, 3801 Miranda Avenue, Palo Alto, CA 94304
- Data Core – Kelly Cho, M.P.H, Ph.D.
  VA Boston Healthcare System, 150 S. Huntington Avenue, Boston, MA 02130
- VA Informatics and Computing Infrastructure (VINCI) – Scott L. DuVall, Ph.D.
  VA Salt Lake City Health Care System, 500 Foothill Drive, Salt Lake City, UT 84148
- Data and Computational Sciences – Saiju Pyarajan, Ph.D.
  VA Boston Healthcare System, 150 S. Huntington Avenue, Boston, MA 02130

- Statistical Genetics – Elizabeth Hauser, Ph.D.

Durham VA Medical Center, 508 Fulton Street, Durham, NC 27705
Yan Sun, Ph.D.
Atlanta VA Medical Center, 1670 Clairmont Road, Decatur, GA 30033
Hongyu Zhao, Ph.D.
West Haven VA Medical Center, 950 Campbell Avenue, West Haven, CT 06516

Current MVP Local Site Investigators

- Atlanta VA Medical Center (Peter Wilson, M.D.)
1670 Clairmont Road, Decatur, GA 30033
- Bay Pines VA Healthcare System (Rachel McArdle, Ph.D.)
10,000 Bay Pines Blvd Bay Pines, FL 33744
- Birmingham VA Medical Center (Louis Dellitalia, M.D.)
700 S. 19th Street, Birmingham AL 35233
- Central Western Massachusetts Healthcare System (Kristin Mattocks, Ph.D., M.P.H.)
421 North Main Street, Leeds, MA 01053
- Cincinnati VA Medical Center (John Harley, M.D., Ph.D.)
3200 Vine Street, Cincinnati, OH 45220
- Clement J. Zablocki VA Medical Center (Jeffrey Whittle, M.D., M.P.H.)
5000 West National Avenue, Milwaukee, WI 53295
- VA Northeast Ohio Healthcare System (Frank Jacono, M.D.)
10701 East Boulevard, Cleveland, OH 44106
- Durham VA Medical Center (Jean Beckham, Ph.D.)
508 Fulton Street, Durham, NC 27705
- Edith Nourse Rogers Memorial Veterans Hospital (John Wells., Ph.D.)
200 Springs Road, Bedford, MA 01730
- Edward Hines, Jr. VA Medical Center (Salvador Gutierrez, M.D.)
5000 South 5th Avenue, Hines, IL 60141
- Veterans Health Care System of the Ozarks (Gretchen Gibson, D.D.S., M.P.H.)
1100 North College Avenue, Fayetteville, AR 72703
- Fargo VA Health Care System (Kimberly Hammer, Ph.D.)
2101 N. Elm, Fargo, ND 58102
- VA Health Care Upstate New York (Laurence Kaminsky, Ph.D.)
113 Holland Avenue, Albany, NY 12208
- New Mexico VA Health Care System (Gerardo Villareal, M.D.)
1501 San Pedro Drive, S.E. Albuquerque, NM 87108
- VA Boston Healthcare System (Scott Kinlay, M.B.B.S., Ph.D.)
150 S. Huntington Avenue, Boston, MA 02130
- VA Western New York Healthcare System (Junzhe Xu, M.D.)
3495 Bailey Avenue, Buffalo, NY 14215-1199
- Ralph H. Johnson VA Medical Center (Mark Hamner, M.D.)
109 Bee Street, Mental Health Research, Charleston, SC 29401

- Columbia VA Health Care System (Roy Mathew, M.D.)
6439 Garners Ferry Road, Columbia, SC 29209
- VA North Texas Health Care System (Sujata Bhushan, M.D.)
4500 S. Lancaster Road, Dallas, TX 75216
- Hampton VA Medical Center (Pran Iruvanti, D.O., Ph.D.)
100 Emancipation Drive, Hampton, VA 23667
- Richmond VA Medical Center (Michael Godschalk, M.D.)
1201 Broad Rock Blvd., Richmond, VA 23249
- Iowa City VA Health Care System (Zuhair Ballas, M.D.)
601 Highway 6 West, Iowa City, IA 52246-2208
- Eastern Oklahoma VA Health Care System (Douglas Ivins, M.D.)
1011 Honor Heights Drive, Muskogee, OK 74401
- James A. Haley Veterans' Hospital (Stephen Mastorides, M.D.)
13000 Bruce B. Downs Blvd, Tampa, FL 33612
- James H. Quillen VA Medical Center (Jonathan Moorman, M.D., Ph.D.)
Corner of Lamont & Veterans Way, Mountain Home, TN 37684
- John D. Dingell VA Medical Center (Saib Gappy, M.D.)
4646 John R Street, Detroit, MI 48201
- Louisville VA Medical Center (Jon Klein, M.D., Ph.D.)
800 Zorn Avenue, Louisville, KY 40206
- Manchester VA Medical Center (Nora Ratcliffe, M.D.)
718 Smyth Road, Manchester, NH 03104
- Miami VA Health Care System (Hermes Florez, M.D., Ph.D.)
1201 NW 16th Street, 11 GRC, Miami FL 33125
- Michael E. DeBakey VA Medical Center (Olaoluwa Okusaga, M.D.)
2002 Holcombe Blvd, Houston, TX 77030
- Minneapolis VA Health Care System (Maureen Murdoch, M.D., M.P.H.)
One Veterans Drive, Minneapolis, MN 55417
- N. FL/S. GA Veterans Health System (Peruvemba Sriram, M.D.)
1601 SW Archer Road, Gainesville, FL 32608
- Northport VA Medical Center (Shing Shing Yeh, Ph.D., M.D.)
79 Middleville Road, Northport, NY 11768
- Overton Brooks VA Medical Center (Neeraj Tandon, M.D.)
510 East Stoner Ave, Shreveport, LA 71101
- Philadelphia VA Medical Center (Darshana Jhala, M.D.)
3900 Woodland Avenue, Philadelphia, PA 19104
- Phoenix VA Health Care System (Samuel Aguayo, M.D.)
650 E. Indian School Road, Phoenix, AZ 85012
- Portland VA Medical Center (David Cohen, M.D.)
3710 SW U.S. Veterans Hospital Road, Portland, OR 97239
- Providence VA Medical Center (Satish Sharma, M.D.)
830 Chalkstone Avenue, Providence, RI 02908
- Richard Roudebush VA Medical Center (Suthat Liangpunsakul, M.D., M.P.H.)

1481 West 10th Street, Indianapolis, IN 46202
- Salem VA Medical Center (Kris Ann Oursler, M.D.)
1970 Roanoke Blvd, Salem, VA 24153
- San Francisco VA Health Care System (Mary Whooley, M.D.)
4150 Clement Street, San Francisco, CA 94121
- South Texas Veterans Health Care System (Sunil Ahuja, M.D.)
7400 Merton Minter Boulevard, San Antonio, TX 78229
- Southeast Louisiana Veterans Health Care System (Joseph Constans, Ph.D.)
2400 Canal Street, New Orleans, LA 70119
- Southern Arizona VA Health Care System (Paul Meyer, M.D., Ph.D.)
3601 S 6th Avenue, Tucson, AZ 85723
- Sioux Falls VA Health Care System (Jennifer Greco, M.D.)
2501 W 22nd Street, Sioux Falls, SD 57105
- St. Louis VA Health Care System (Michael Rauchman, M.D.)
915 North Grand Blvd, St. Louis, MO 63106
- Syracuse VA Medical Center (Richard Servatius, Ph.D.)
800 Irving Avenue, Syracuse, NY 13210
- VA Eastern Kansas Health Care System (Melinda Gaddy, Ph.D.)
4101 S 4th Street Trafficway, Leavenworth, KS 66048
- VA Greater Los Angeles Health Care System (Agnes Wallbom, M.D., M.S.)
11301 Wilshire Blvd, Los Angeles, CA 90073
- VA Long Beach Healthcare System (Timothy Morgan, M.D.)
5901 East 7th Street Long Beach, CA 90822
- VA Maine Healthcare System (Todd Stapley, D.O.)
1 VA Center, Augusta, ME 04330
- VA New York Harbor Healthcare System (Scott Sherman, M.D., M.P.H.)
423 East 23rd Street, New York, NY 10010
- VA Pacific Islands Health Care System (George Ross, M.D.)
459 Patterson Rd, Honolulu, HI 96819
- VA Palo Alto Health Care System (Philip Tsao, Ph.D.)
3801 Miranda Avenue, Palo Alto, CA 94304-1290
- VA Pittsburgh Health Care System (Patrick Strollo, Jr., M.D.)
University Drive, Pittsburgh, PA 15240
- VA Puget Sound Health Care System (Edward Boyko, M.D.)
1660 S. Columbian Way, Seattle, WA 98108-1597
- VA Salt Lake City Health Care System (Laurence Meyer, M.D., Ph.D.)
500 Foothill Drive, Salt Lake City, UT 84148
- VA San Diego Healthcare System (Samir Gupta, M.D., M.S.C.S.)
3350 La Jolla Village Drive, San Diego, CA 92161
- VA Sierra Nevada Health Care System (Mostaqul Huq, Pharm.D., Ph.D.)
975 Kirman Avenue, Reno, NV 89502
- VA Southern Nevada Healthcare System (Joseph Fayad, M.D.)
6900 North Pecos Road, North Las Vegas, NV 89086

- VA Tennessee Valley Healthcare System (Adriana Hung, M.D., M.P.H.)
1310 24th Avenue, South Nashville, TN 37212
- Washington DC VA Medical Center (Jack Lichy, M.D., Ph.D.)
50 Irving St, Washington, D. C. 20422
- W.G. (Bill) Hefner VA Medical Center (Robin Hurley, M.D.)
1601 Brenner Ave, Salisbury, NC 28144
- White River Junction VA Medical Center (Brooks Robey, M.D.)
163 Veterans Drive, White River Junction, VT 05009
- William S. Middleton Memorial Veterans Hospital (Robert Striker, M.D., Ph.D.)
2500 Overlook Terrace, Madison, WI 53705
